# Emerging Contaminants in Source and Finished Drinking Waters Across Minnesota (U.S.) and Potential Health Implications

**DOI:** 10.3390/ijerph22070976

**Published:** 2025-06-20

**Authors:** Sarah M. Elliott, Aliesha L. Krall, Jane R. de Lambert, Maya D. Gilchrist, Stephen W. Robertson

**Affiliations:** 1 U.S. Geological Survey, 2280 Woodale Drive, Mounds View, MN 55112, USA; akrall@usgs.gov; 2 Minnesota Department of Health, 625 Robert Street North, Saint Paul, MN 55155, USA; jane.de.lambert@state.mn.us (J.R.d.L.); maya.gilchrist@state.mn.us (M.D.G.); steve.robertson@state.mn.us (S.W.R.)

**Keywords:** pharmaceuticals, PFAS, pesticides, surface water, groundwater, public water supply, human health

## Abstract

Relatively little data exist regarding the presence of unregulated contaminants in drinking waters. We sampled source and finished drinking water from 98 community water supply systems throughout Minnesota (U.S.). Facilities were grouped into four networks based on water source and influences from anthropogenic activities. Measured contaminants were dependent on network and included some combination of pesticides, pharmaceuticals, per- and poly-fluoroalkyl substances (PFAS), benzotriazoles, hormones, wastewater indicators, and illicit drugs. Overall, the number of contaminants detected in samples ranged from 0 to 35 and concentrations ranged from 0.38 ng/L (progesterone) to 47,500 ng/L (bromoform). Fewer contaminants and lower concentrations were detected in finished water samples, compared to source waters. Significantly (*p* < 0.05) more PFAS and pesticides and higher sample total concentrations were observed in wells designated as vulnerable to contamination. To estimate potential human-health risk from exposure in drinking water, concentrations were compared against bioactivity information from the U.S. Environmental Protection Agency’s ToxCast database and state-based guidance values, when available. Although comparisons could be made for relatively few contaminants, concentrations in finished waters were at least an order of magnitude lower than screening thresholds. Results from this study were used to inform enhancement of the Minnesota Department of Health’s drinking water protection program.

## 1. Introduction

Globally, the presence of human-derived contaminants in aquatic environments has been well documented [1,2,3]. With tens of thousands of contaminants in use today (plus their transformation products) it is difficult to quantify the potential human-health effects resulting from environmental exposure. However, many environmental contaminants are known or suspected endocrine disruptors and/or have health implications such as increased risk of cancer, impaired metabolism, or other sub-lethal effects [4]. Following guidance from the U.S. Environmental Protection Agency’s Safe Drinking Water Act [5], treatment of drinking water and compliance monitoring are conducted to ensure safe drinking water is available to the public. However, monitoring is typically focused on regulated contaminants (e.g., nitrate, arsenic) and has not kept pace with the production of other contaminants [6]. Currently, there are limited data on the occurrence, magnitude, and toxicology of unregulated contaminants, and the factors (hydrogeologic, hydrologic, treatment, etc.) that are related to exposure and risk in drinking water [7].

In the United States (U.S.), both groundwater and surface water are used as sources of drinking water, and most states have direct oversight of their community water supply systems. Anthropogenic activities on the landscape affect the quality of both groundwater and surface water resources used as drinking water, although the risk profile for each resource may differ [8,9]. Surface water quality is often directly influenced by discharge from wastewater treatment plants, hospitals, and industrial facilities [10]. Because treatment methods are not designed to remove contaminants such as pharmaceuticals, personal care products, surfactants, and flame retardants, they are often introduced to receiving streams [11,12]. Furthermore, non-point sources of contaminants such as runoff from urban and/or agricultural areas can introduce pesticides and other contaminants that have built up on the land surface into receiving streams [13,14,15]. Although groundwater may be somewhat buffered from anthropogenic activities on the landscape, contaminants can be introduced to groundwater resources via landfill leachates, septic effluents, and other infiltration practices [16,17,18], resulting in similar contaminant profiles as are seen in surface waters. Given these varied sources of potential contamination to surface and groundwaters, it is important to understand how each is affected and the implications for drinking water supplies.

Several studies have reported the occurrence of emerging contaminants (e.g., pharmaceuticals, personal care products, current-use pesticides) in source and finished (i.e., treated) drinking waters across the globe, with greater presence in surface water sources compared to groundwater [3,9,19]. However, even when the number of contaminants analyzed reaches into the hundreds, a relatively small percentage is detected [9,20]. A study of pharmaceuticals and hormones in groundwater used as drinking water supply across the U.S. revealed low occurrence of both types of contaminants (at least one detected in 7% of sites) and concentrations below human-health benchmarks; detections were related to well depth, age of water, and aquifer type [21]. Similar results were reported for various anthropogenic contaminants in groundwater drinking water sources in Milan, Italy [22]. Community public supply facilities employ targeted treatments for specific goals with a focus on regulated contaminants. Although incidental removal of some emerging contaminants occurs, many are not removed and removal is dependent on the treatment employed and the physicochemical properties of individual contaminants [23,24]. For many of these contaminants, guidance or other screening values do not exist, preventing full assessment of risk to exposure [25,26]. Collection of data related to the presence of emerging contaminants in drinking waters can help prioritize contaminants for development of health screening values to better assess risks to human health from both regulated and unregulated/unmonitored contaminants.

Minnesota (U.S.) has 975 active community water supply systems. Although some previous studies (e.g., [20,27]) have characterized the presence of emerging (and mostly unregulated) contaminants, these studies have generally been small in scope and related to specific project objectives. No broad-scale survey has been conducted in Minnesota to characterize the presence of unregulated contaminants in the state’s drinking water supplies. The overall objective of this study was to fill data gaps related to the presence of emerging contaminants (most of which are unregulated) in public-supply source and finished drinking waters throughout Minnesota. To reach that objective, two goals were identified: (1) characterize the presence and magnitude of emerging contaminants in source and finished drinking waters and (2) compare concentrations of emerging contaminants against available health-based guidance values to assess the potential health risk to Minnesota residents. The results from our study provide the first comprehensive assessment of unregulated contaminants in Minnesota’s drinking waters. The results expand on other studies by considering the type of source water, potential contaminant sources within drinking water source management areas, and likelihood of contamination for groundwater facilities. Additionally, results were used to inform development of a routine monitoring program focused on unregulated contaminants and to inform the development of guidance values for drinking water.

## 2. Materials and Methods

**Figure 1:**
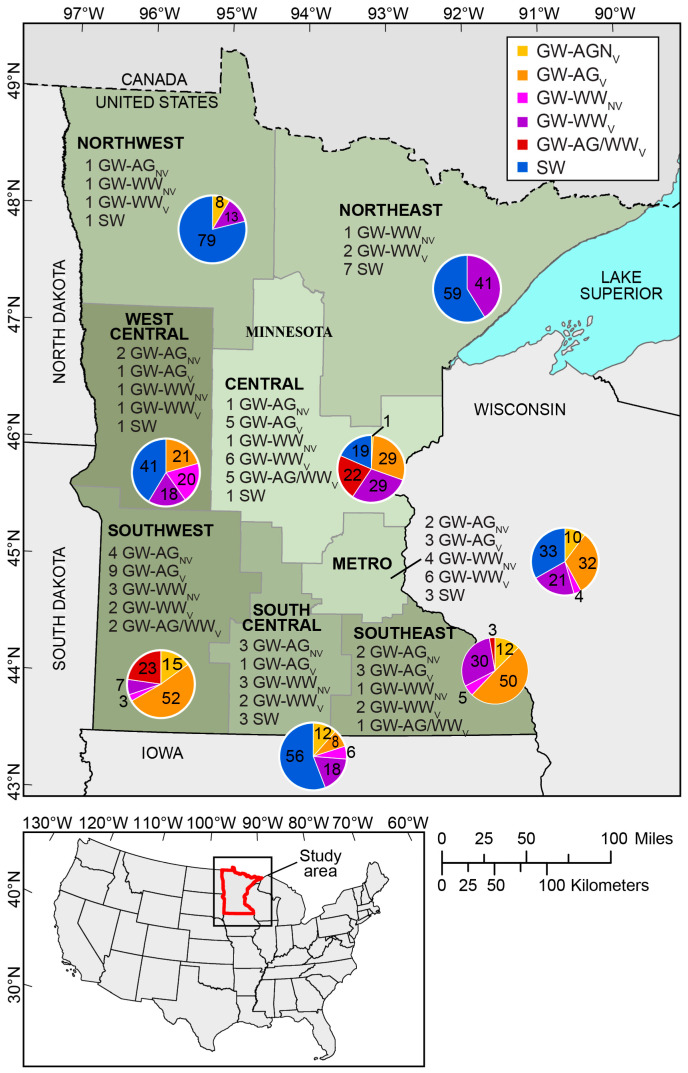
Map showing general locations of Minnesota community water supply systems from which paired source and finished water samples were collected, 2019–2022. Pie charts show the percentage of unique contaminants detected in each sampling network located within each region. Community water supply systems were categorized by facility type: surface water sourced (SW), groundwater sourced likely to be influenced by agricultural activities on the landscape (GW-AG), groundwater sourced likely to be influenced by wastewater inputs to the environment (GW-WW), and groundwater sourced likely to be influenced by both agricultural activities and wastewater inputs to the environment (GW-AG/WW). The number of water supply systems sampled is provided in front of the facility type designation. Subscripts ‘V’ and ‘NV’ indicate if the groundwater well was considered vulnerable or non-vulnerable to pollution, respectively. Basemap from U.S. Geological Survey 1:24,000 2023 National Boundary Dataset; Universal Transverse Mercator; zone 15 North; North American Datum of 1983.

A total of 98 community water supply facilities (~10% of all facilities in Minnesota) voluntarily participated in the study (Figure 1, Table S1). The types of contaminants that were analyzed at each facility varied by network and well classification, as described in Section 2.1 (Tables S2 and S3). A total of 82 facilities represented groundwater sources (9% of groundwater facilities in Minnesota), the primary source of drinking water in Minnesota, while 16 facilities represented surface water sources (70% of surface water facilities in Minnesota). Facilities were categorized into networks based on water source type and/or expected contaminant sources influenced by land use: surface water (SW), groundwater facilities likely to be influenced by agricultural activities on the landscape (GW-AG), groundwater facilities likely to be influenced by wastewater inputs to the environment (GW-WW), and groundwater facilities likely to be influenced by both agricultural activities and wastewater inputs (GW-AG/WW). Although water quality of surface waters may also be influenced by different activities on the landscape, we chose to keep surface water-sourced facilities in one group and analyze samples for all contaminants since they are more directly influenced by activities on the landscape.

Groundwater facilities were further classified as vulnerable or non-vulnerable, which refers to the likelihood that activities on the land surface may impact drinking water quality. Vulnerability is determined using multiple factors, including geologic sensitivity, chemical and isotopic evidence of recent recharge, and well construction and maintenance [28]. Geologic sensitivity accounts for the ability of the earth’s geologic materials to protect a well from contaminant sources and is inversely proportional to the travel time required for water to move vertically from the land surface to an aquifer. Water chemistry is used to identify the presence or absence of contaminants, and isotopic signatures are used to estimate recharge rates. Generally, a facility is classified as vulnerable if the well is subject to recent recharge and potential effects of land use. Conversely, a facility is classified as non-vulnerable if it is isolated from potential effects of land use by geologic conditions that limit groundwater recharge. Given the lack of potential influence from activities on the landscape and potential for contamination at the non-vulnerable wells, these facilities can be considered as a type of control.

### 2.1. Site Selection

#### 2.1.1. Surface Water Network Facilities

Of the 23 community water systems that rely on surface water in Minnesota, 16 (70%) were included in the study. In instances where several facilities rely on the same source (e.g., Lake Superior, Red Lake River), only one facility was selected for inclusion in the study to reduce redundancy and maximize resources. Because surface water is vulnerable to various contaminant sources (e.g., wastewater effluent, stormwater runoff, industrial discharge), a broad suite of contaminants was analyzed, including pharmaceuticals and personal care products, contaminants indicative of anthropogenic influence (i.e., wastewater indicators), per- and polyfluoroalkyl substances (PFAS), illicit drugs, hormones, and pesticides and pesticide degradates (hereafter referred to as ‘pesticides’) (Table 1 and Table S2).

#### 2.1.2. Groundwater Network Facilities

A total of 37 facilities likely to be influenced by agricultural activities were sampled as part of the GW-AG network. Facilities for which land use within the well’s drinking water supply management area (DWSMA) was predominately agricultural and for which there was evidence of elevated nitrate (>3 mg/L), presence of pesticides, and/or presence of livestock specific pathogens in groundwater were included in the GW-AG network. An additional 37 facilities were identified for inclusion in the GW-WW network. These facilities were determined to be likely influenced by wastewater inputs to the environment. based on previous water quality data and the presence of point sources. Available water quality results indicating chloride to bromide ratios between 500 and 800, chloride concentrations between 40 and 100 mg/L, and the presence of pharmaceuticals or artificial sweeteners were used as indicators of wastewater influence on groundwater. Additionally, the presence of wastewater treatment plants, subsurface sewage treatment systems, permitted discharges, and sewer infrastructure were considered. Within each network, sites were ranked based on these criteria and those with the highest ranks were selected for inclusion in the study. Samples collected from GW-AG facilities were analyzed for pesticides and PFAS. Samples collected from GW-WW facilities were analyzed for wastewater indicators (vulnerable only), pharmaceuticals and personal care products, PFAS, benzotriazoles, alkylphenols, and pesticides. Non-vulnerable wells were sampled for additional benzotriazoles, compared to vulnerable wells (Table 1). Of the groundwater facilities, 22 in each of the GW-AG and GW-WW networks were designated as vulnerable, and 15 as non-vulnerable.

A total of eight facilities were located in areas that are potentially influenced by both agricultural activities and wastewater inputs to the environment. Samples collected from these sites were analyzed for the same contaminants as GW-AG and GW-WW combined. These sites are represented in a separate network (GW-AG/WW) to highlight differences between groundwater facilities with one potential type of contaminant source (e.g., agriculture) and those with multiple potential sources.

### 2.2. Sample Collection and Analysis

Paired source and finished drinking water samples were collected during 2019 to 2022. Groundwater facilities were sampled once during August 2019 to March 2022. Six groundwater facilities did not employ any treatment to source waters, so the unpaired sample was included as both a source and finished water sample in summaries. Surface water facilities were sampled twice during August 2019 to November 2019 to capture some variability associated with contaminant presence in surface waters. Source water samples were collected at the well or surface water intake and finished samples were collected from the entry point at which the finished drinking water enters the distribution system. Source and finished water samples were collected consecutively on the same day, so sample pairs do not represent the same parcel of water. Water lines were flushed for 15 min and physical water properties (temperature, dissolved oxygen, pH, specific conductivity) were allowed to stabilize prior to sample collection. For samples intended for analysis of pesticides and pharmaceuticals at the U.S. Geological Survey National Water Quality Laboratory (NWQL), water was filtered through a 0.7 µm syringe-tip filter in the field. All other samples were collected as whole-water samples. A total of eight field blank and seven field replicate samples were collected to assess potential contamination and variability associated with sample collection and processing, respectively. Samples were stored chilled or frozen, depending on targeted analysis, until shipment to the analyzing laboratory. Details regarding analytical methods, reporting levels, and other relevant information can be found in Table S2.

### 2.3. Data Preparation and Analysis

Some contaminants were included in multiple analytical methods (e.g., atrazine was included in the pharmaceutical, wastewater indicator, and pesticide methods). For instances in which there was more than one value for a contaminant in a sample, the value associated with the more sensitive method (i.e., lower reporting level) was retained for analysis. Some exceptions were the triazine pesticides atrazine, deethylatrazine, deisopropylatrazine, didealkylatrazine, hydroxyatrazine, and cyanazine, for which NWQL results were retained over others. Three contaminants were detected in at least one field blank sample (Table S4). Environmental sample concentrations <10 times the maximum field blank concentration were removed from the dataset for analysis. This affected 21 norgestrel, 8 nicosulfuron, and 3 tris(2-butoxyetheyl) phosphate values. A total of 207 values from 42 contaminants and 59 samples were assigned a code by the analyzing laboratory indicating potential lab contamination and were removed from the dataset for analysis. Lastly, because of quality control concerns at the analyzing laboratory, all mestranol values were removed from the dataset. With respect to field-replicate samples, when a contaminant was detected in both samples of a pair, the relative percent difference (RPD) was calculated by dividing the absolute difference between the concentrations by the average concentration and multiplying by 100. Overall, RPD ranged from 0 to 67%, with most comparisons (82%) below 25% (Tables S5 and S6).

All statistical analyses were completed in R (v.4.4.1; [29]). Summary statistics including the detection frequency, minimum concentration, and maximum concentration were calculated. For each sample, the total number of contaminants detected and total concentration of each contaminant group were calculated. For total contaminant group concentration calculations, individual contaminant concentrations within each group were summed; values reported as a non-detect (i.e., less than the reporting level) were considered to be zero. Differences (centroids and dispersion) in the total number of contaminant detections and total concentrations among source water samples representing different networks and/or well classifications were assessed by one-way permutational multivariate analysis of variance (PERMANOVA) on Euclidean distance using the vegan package (v. 2.6-6.1; [30]). Differences between paired source and finished drinking water samples, by network and contaminant group, were assessed by the Wilcoxon signed-rank sum test with the Pratt adjustment using the NADA2 package (v. 1.1.6; [31]). Because of the limited number of detections, no statistical comparisons were completed for alkylphenols, hormones, or illicit drugs. Additionally, the number of benzotriazole detections from groundwater-sourced facilities was limited, so statistical comparisons could be assessed only for surface water-sourced facilities.

### 2.4. Human-Health Risk Screening

We used two screening methods to prioritize contaminants of potential concern and assess potential risks associated with exposure to contaminants in finished drinking water samples. First, we compared detected concentrations to bioactivity data from the U.S. Environmental Protection Agency’s ToxCast database (v. 4.1) using the toxEval package (v. 1.4; [32]). The ToxCast database contains bioactivity information obtained from molecular assays for thousands of chemicals as they relate to effects on cells, receptors, etc. For each chemical–endpoint match, an exposure activity ratio (EAR) was calculated by dividing the environmental concentration by the activity concentration at cutoff (ACC). The ACC defines a threshold above which biological activity is expected to occur and is standardized to a response threshold, so is often favored over other metrics [33]. Maximum EAR values were determined for each contaminant detected within a sample and maximum values were summed to obtain an estimate of the potential cumulative risk (∑EAR_max_). For the surface water facilities at which two samples were collected, the maximum ∑EAR_max_ at each site was used in analysis. We used a threshold of 0.001 to identify individual contaminants that may warrant some level of concern for human health, as has been used by others (e.g., [20,34]). PFAS were excluded from this screening because relatively few of the detected (1 of 15) PFAS were represented in the database, which would grossly underestimate potential risk. We opted to use the state-based health guidance values to screen PFAS data since they are available for more compounds.

In addition to the molecular approach, we used state-based health guidance values and/or rapid screening values (hereafter collectively referred to as ‘guidance values’) used by the Minnesota Department of Health (MDH) to assess potential risk. A total of 211 guidance values were available (Table S2), 99 of which were applicable to contaminants detected in environmental samples. Similar to the EAR approach, we divided environmental concentrations by guidance values to obtain a toxicity quotient (TQ). In instances when more than one guidance value was available, we used the minimum. We used a threshold of 0.1 to identify individual contaminants that may pose a greater risk than others.

These screening methods were applied to provide context for the environmental data and likely do not capture true risk because of limitations such as available analytical methods and available screening values. Furthermore, it is important to note that although EAR screening can provide contextual information for environmental concentrations, not all identified effects are negative and there is uncertainty associated with translation of the identified molecular effects to human exposure. Despite limitations, these results can provide valuable information related to prioritization of contaminants for future monitoring, nomination to the MDH risk screening program, and/or other research.

## 3. Results and Discussion

**Figure 2:**
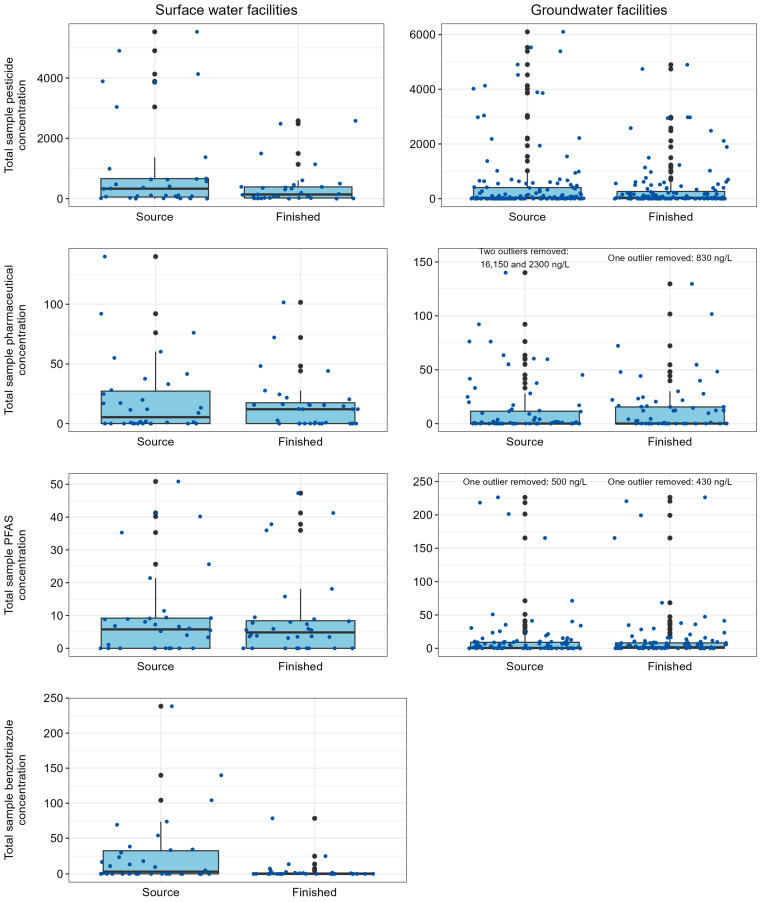
Boxplot summaries of total sample contaminant concentrations (ng/L), by group, in source and finished water samples collected from community water facilities in Minnesota, 2019–2022. Individual samples are represented by dark blue dots. The 25th, 50th, and 75th percentiles are represented by the bottom of the box, black line within the box, and top of the box, respectively. Whiskers extend to the minimum and maximum values and outliers are represented by black dots. PFAS, per- and polyfluoroalkyl substances.

At least one contaminant was detected in source and finished water samples at 87 (90%) of the sampled facilities. Of the 513 contaminants that were analyzed, 170 (33%) were detected in at least one sample. However, 127 (75%) of the detected contaminants were observed in <10% of samples. Excluding bromoform, individual concentrations ranged from 0.384 (progesterone) to 21,600 (isophorone) ng/L, with most (87%) concentrations < 100 ng/L. Bromoform concentrations ranged from 10 to 47,500 ng/L (Figure 2, Table S7). The number of contaminants detected in each sample ranged from 1 (several source and finished) to 77 (surface water source), excluding the 12 facilities at which none were detected. Pesticide transformation products represented most of the contaminants that were detected in ≥25% of samples. No clear patterns in relations between select explanatory variables (e.g., number of permitted dischargers, population served by the facility, types of permitted dischargers) and number of contaminants detected or concentrations detected were observed for this study.

### 3.1. Contaminant Presence in Source and Finished Drinking Waters

#### 3.1.1. Surface Water Network

Samples collected from the SW network were analyzed for the broadest suite of contaminants (*n* = 502) among all the networks. A total of 117 and 74 contaminants were detected in source and finished water samples, respectively, while the number of contaminants detected in individual samples ranged from 6 to 77. Excluding bromoform, concentrations ranged from 0.384 (progesterone) to 5400 (cholesterol) ng/L in source water samples and from 0.502 (roxithromycin) to 21,600 (isophorone) ng/L in finished water samples (Table S7). Bromoform, a disinfection byproduct produced during the treatment process, was detected in all finished water samples at concentrations up to 47,500 ng/L. Although more contaminants were detected in ≥25% of source water samples, compared to finished, similar detection frequencies were observed between sample types for several contaminants (e.g., atrazine, metolachlor, deethylatrazine, PFBA, metformin), suggesting little to no removal of these contaminants during the treatment process.

The number of pesticides detected and total pesticide concentrations in individual samples were greater in source water compared to finished water (*p* < 0.001; Table S8, Figure S1). A total of 53 (24% of those analyzed) and 33 (15%) were detected in source and finished water samples, respectively, though most were detected in <5 samples. Facilities located in agricultural dominated areas tended to have more pesticides detected compared to those located near urban or forested areas. The highest concentrations were observed for the transformation products metolachlor SA (772 ng/L) and acetochlor OA (882 ng/L). Overall, atrazine was the most frequently detected (86%) pesticide in all samples and is also one of the most frequently detected pesticides in surface waters throughout Minnesota and the U.S. [35,36,37]. This agrees with studies that have shown positive relations between atrazine and corn acreage [38], since Minnesota is one of the top corn producers in the U.S. Furthermore, atrazine has been designated a ‘surface water pesticide of concern’ by the Minnesota Department of Agriculture, which is determined based on concentrations of concern relative to a water quality reference value [37]. Atrazine was detected in 84% of finished water samples, similar to surface water sourced drinking water in other parts of the world [39,40,41].

Overall, there was no significant difference in total concentrations or number of detected PFAS between paired source and finished water samples (*p* > 0.05; Table S8; Figure S1). Similar results were reported in a nationwide study of PFAS in source and treated drinking waters [6]. In most finished water samples, ≤5 PFAS were detected. However, >10 PFAS were detected at two facilities. Maximum concentrations of individual PFAS ranged from 0.943 (PFNA) to 33.2 (PFBA) ng/L, with most <5 ng/L. Generally, these concentrations were at least an order of magnitude lower than mean and maximum concentrations of six PFAS (not including PFBA) in finished water samples collected from large surface-water sourced systems in the U.S. [42]. Although PFAS concentrations are not dependent on the size of a water body, generally, higher concentrations of PFAS are observed in larger water bodies due to the larger surface area and therefore a greater capacity to accumulate contaminants from sources through industrial and wastewater discharges and/or runoff from contamination sites. Total PFAS concentrations ranged from 3.13 to 47.29 ng/L in individual samples. Concentrations observed in our study are relatively low compared to those reported by the USEPA (3rd Unregulated Contaminant Monitoring Rule) and across the U.S., but some PFAS in the USEPA study could be underreported because of high reporting limits [42]. PFBA was the most frequently detected (67%) PFAS in all samples with the greatest maximum concentration (33.2 ng/L) in finished water. PFBA was also the most frequently detected PFAS in finished and tap water samples collected from a subset of facilities within the Minneapolis-St. Paul area [20]. Similarly, PFBA was detected in all finished water samples analyzed in the Netherlands representing about 1/3 of the nation’s surface water sourced facilities [43]. The relatively low concentrations of PFAS observed during our study are not indicative of major dischargers and suggest that contamination is likely from diffuse sources within the watershed such as different water treatment plants and/or stormwater runoff, as has been observed in other studies [44,45]. Although our study did not observe concentrations of concern, regular monitoring would be important to identify potential changes and/or accidents from dischargers.

Overall, few pharmaceuticals (≤1%) were detected. This is notable because as integrators of regional wastewater streams, wastewater treatment effluents are major contributors of pharmaceuticals to receiving surface waters since they are not designed to treat these contaminants effectively. However, despite more pharmaceutical detections in source water samples (*p* < 0.1), similar total concentrations were observed in source and finished water samples (*p* > 0.1; Table S8; Figure S1). Four (2%) or fewer pharmaceuticals were detected in finished water samples at concentrations ranging from 0.502 ng/L (roxithromycin) to 86.2 ng/L (desmethyldiltiazem). The low number of pharmaceuticals detected in our samples is similar to a study restricted to the Minneapolis-St. Paul area of Minnesota [20], studies conducted throughout the U.S. [46,47], and a nationwide study [48], but our maximum concentrations were generally slightly lower. Furlong et al. [48] reported that caffeine and carbamazepine were detected in >50% of finished samples from surface water sourced facilities. However, we did not detect either pharmaceutical in any finished water samples. Metformin was, by far, the most frequently detected pharmaceutical in finished water samples (30%). This is higher than what has been reported in other studies in the U.S. and across the globe [20,48,49,50].

The presence and total concentrations of benzotriazoles and wastewater indicators were significantly greater in source water samples, compared to finished (*p* < 0.05 and *p* < 0.001, respectively). Bromoform concentrations were significantly greater in finished water samples (*p* < 0.001; Table S8, Figure S1). As mentioned previously, bromoform is a common byproduct of disinfection with chlorine, which is necessary to remove harmful pathogens from drinking water and is currently regulated. Total benzotriazole concentrations in finished water samples were substantially lower than those reported in tap waters in China [51] and tap waters in South Korea [52]. Furthermore, Wang et al. [52] reported efficient removal of benzotriazoles, in particular by adsorption processes.

#### 3.1.2. Groundwater Sourced Facilities Likely to Be Influenced by Agricultural Activities on the Landscape

Samples collected from the GW-AG network were analyzed for PFAS and pesticides. A total of 45 and 43 contaminants were detected in source and finished water samples, respectively, while the number of contaminants detected in each sample ranged from 1 to 15. Concentrations ranged from 0.403 (PFHxS) to 4910 (metolachlor SA) ng/L in source water samples and from 0.387 (PFOSA) to 2510 (alchlor SA) ng/L in finished water samples. In source water samples, four contaminants were detected in >25% of samples, including three pesticides (atrazine, deethylatrazine, and metolachlor SA) and PFBA. Comparatively, nine contaminants were detected in ≥25% of finished water samples, including four pesticides (atrazine, deethylatrazine, metolachlor SA, and deethylcyanazine acid) and five PFAS (PFBA, PFBS, PFOSA, PFHxS, and PFHxA).

##### Vulnerable Groundwater Wells Likely to Be Influenced by Agricultural Activities on the Landscape

No significant difference in total concentrations or number of pesticides detected were observed between source and finished water samples collected from vulnerable GW-AG wells (*p* > 0.05 and *p* > 0.01, respectively; Table S8). Among finished water samples, 0 to 15 pesticides were detected. Maximum concentrations of pesticides in finished water samples ranged from 1.04 (propazine) to 2510 ng/L (alachlor SA). Metolachlor SA was the most frequently detected pesticide (80%) in finished water samples and had the second highest maximum concentration (2470 ng/L). Metolachlor SA and atrazine were also reported as frequently detected herbicides in groundwater used for public water supply across the U.S. [53]. The top five pesticides with the highest maximum concentrations were transformation products (Table S7). As pesticides are applied to the landscape, they may migrate to groundwater resources via precipitation and irrigation, although they may be degraded or transformed microbially or physically in the process.

More PFAS and greater total sample concentrations were observed in finished water samples compared to source (*p* < 0.01; *p* < 0.05; Table S8; Figure S2). For PFAS, 0 to 12 compounds were detected in source and finished drinking water samples. Of those detected, >10% were detected in finished water samples. More than five PFAS were detected in finished water samples collected from seven (32%) facilities. Concentrations of individual PFAS ranged from 0.387 (PFOSA) to 436 (PFBA) ng/L. The total sample concentrations of two finished water samples were >200 ng/L, an order of magnitude higher than the other samples because of high concentrations of PFBA. Overall, patterns in the number of PFAS detected and total concentration were similar between source and finished water samples, as has been observed in other studies [33,42]. When considering paired source and finished water samples, the number of PFAS detected and total concentration were significantly greater in finished water samples (*p* < 0.05; Table S8, Figure S2). This difference was driven by PFOSA, which was detected in six finished water samples but not the paired source sample, and PFBA, which was detected at relatively high concentrations. PFOSA is a precursor to PFOS, so it might be expected that presence and concentrations of PFOSA would be lower in finished drinking water samples compared to source. However, Xiao et al. [54] observed more reactivity of polyfluoroalkyl sulfonamides with ozonation compared to chlorination. When total PFAS concentrations in finished water samples were greater than source, the difference ranged from 0.1 to 19 ng/L. These differences could be attributed to the fact that the same parcel of water was not sampled and/or that precursors not included in the study were present in source waters.

##### Non-Vulnerable Groundwater Wells Likely to Be Influenced by Agricultural Activities on the Landscape

**Figure 3:**
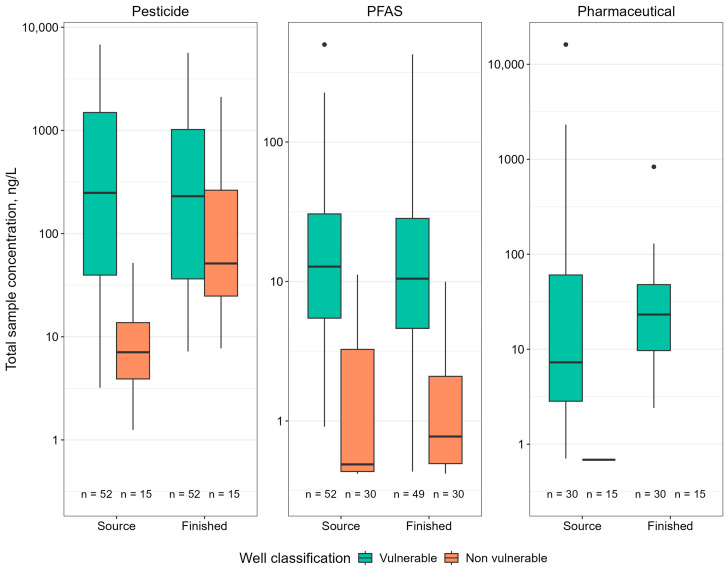
Boxplot summary of total sample concentrations of pesticides, per- and polyfluoroalkyl substances (PFAS), and pharmaceuticals detected in source and finished water samples collected from groundwater-sourced drinking water facilities classified as vulnerable and non-vulnerable. No pharmaceuticals were detected in finished water samples collected from non-vulnerable wells. The 25th, 50th, and 75th percentiles are represented by the bottom of the box, black line within the box, and top of the box, respectively. Whiskers extend to the minimum and maximum values and outliers are represented by individual dots. Number of samples are indicated on the bottom of each boxplot.

Overall, few pesticides and PFAS were detected (≤1 to 3%) in samples collected from the seven non-vulnerable GW-AG wells (Table S7). No significant difference in the PFAS and pesticide total concentration or number detected were observed between source and finished water samples (*p* < 0.05; Figure 3 and Figure S3, Table S8). Sixteen pesticides were detected in source and finished drinking water samples. The number of pesticides detected in finished water samples ranged from zero to seven, with concentrations ranging from 0.62 (propoxur) to 1340 (alachlor SA) ng/L. No pesticides were detected in 64% of finished water samples. Metolachlor SA was the most frequently detected (21%) pesticide in finished water samples. Furthermore, nine PFAS were detected in finished water samples. The number of individual PFAS detections ranged from zero to seven in finished water samples, with concentrations ranging from 0.39 (PFHxA) to 6.63 (6:2 FTS) ng/L. No PFAS were detected in 50% of finished water samples. PFOSA was the most frequently detected PFAS in finished water samples (36%). Although no difference was observed between PFAS in source and finished drinking water, PFOSA was often observed in finished samples and not detected in the paired source water sample, similar to the vulnerable GW-AG wells.

#### 3.1.3. Groundwater Sourced Facilities Likely to Be Influenced by Wastewater Inputs to the Environment

Samples collected from the GW-WW network were analyzed for PFAS, wastewater indicators, and pharmaceuticals. A total of 39 and 41 contaminants were detected in source and finished water samples, respectively. The number of contaminants detected in each sample ranged from 0 to 14. Out of the 12 most frequently detected (>20%) contaminants, 7 were PFAS (Table S7). Excluding bromoform, maximum concentrations ranged from 0.418 (PFOSA) to 15,900 (gabapentin) ng/L in source waters and from 0.419 (PFOSA) to 4000 [bis(2-ethylhexyl)phthalate] ng/L in finished water samples. Bromoform concentrations ranged from 10 to 20 ng/L in source water samples and from 20 to 40,900 ng/L in finished water samples. Aside from bromoform, which was significantly greater in finished water samples compared to source (*p* < 0.001; Table S8), no significant differences in the number of contaminants detected or total sample concentrations were observed between source and finished water samples for PFAS, wastewater indicators, and pharmaceuticals (*p* > 0.05; Table S8; Figures S4 and S5).

##### Vulnerable Groundwater Wells Likely to Be Influenced by Wastewater Inputs to the Environment

Five contaminants were detected in ≥25% of source water samples, including four PFAS (PFBA, PFBS, PFHxS and PFOA), sulfamethoxazole, and atrazine. Comparatively, six contaminants were detected in ≥25% of finished water samples, including bromoform, four PFAS (PFBA, PFBS, PFHxS, and PFOA), and atrazine. The high occurrence frequency of atrazine in groundwater is consistent with the surface water facilities, highlighting the highly mobile properties of this pesticide [55]. Excluding bromoform, maximum concentrations ranged from 0.707 (sulfamethoxazole) to 600 (pentachlorophenol) ng/L in source waters and from 0.791 (PFHxA) to 4000 [bis(2-ethylhexyl)phthalate] ng/L in finished water samples. Bromoform concentrations ranged from 10 to 20 ng/L in source water samples and from 20 to 40,900 ng/L in finished water samples.

Most detected pharmaceuticals and wastewater indicators were detected in <10% of finished water samples (Table S7). Of the 13 (<1% of those analyzed) detected pharmaceuticals, maximum concentrations in finished water samples were <20 ng/L, except for acetaminophen (796 ng/L), desmethyldiltiazem (47 ng/L), diatrizoic acid (35 ng/L), and colchicine (23 ng/L). Wastewater indicators that were detected in finished water samples were observed in <3% of samples. Most (63%) individual wastewater indicators detected were detected in a single sample. The highest concentrations for wastewater indicators were observed for bis(2-ethylhexyl) phthalate (4000 ng/L), tri(2-butoxyethyl) phosphate (1970 ng/L), and pentachlorophenol (500 ng/L). These results indicate that typical sources of pharmaceuticals and other contaminants associated with permitted dischargers such as wastewater treatment plants and/or subsurface treatment systems are influencing groundwater quality to some degree.

##### Non-Vulnerable Groundwater Wells Likely to Be Influenced by Wastewater Inputs to the Environment

Few contaminants were detected in samples collected from non-vulnerable GW-WW facilities. Diphenhydramine was the only pharmaceutical detected (0.686 ng/L in one source water sample) and benzothiazole was the only benzotriazole detected (180 ng/L in one finished water sample). The maximum number of PFAS detected in source water samples was one, while in finished water samples it was two (Table S7). Three PFAS were detected at nine facilities, including PFOSA, 6:2 FTS, and MeFOSAA. Both 6:2 FTS (7.76 ng/L) and MeFOSAA (1.69 ng/L) were detected in a single source and finished water sample, respectively. PFOSA was the most frequently detected PFAS with a maximum concentration of 1.58 ng/L and was detected in more finished than source water samples, as observed in the GW-AG network.

#### 3.1.4. Groundwater Sourced Facilities Likely to Be Influenced by Both Agricultural Activities on the Landscape and Wastewater Inputs to the Environment

Because of potential influence from agriculture and wastewater sources, eight facilities are presented individually as the GW-AG/WW network. All facilities in this network were classified as vulnerable. Generally, results were similar to those observed from the individual vulnerable agriculture and wastewater facilities. Samples collected from the GW-AG/WW were analyzed for the same target contaminants as vulnerable GW-WW facilities, plus pesticides (Table 1). Source and finished water sample results were not statistically compared because of insufficient sample size. Up to 25 and 22 contaminants were detected in source and finished water samples, respectively, with maximum concentrations of 2040 ng/L (gabapentin) and 515 ng/L (metolachlor SA), excluding bromoform. Maximum concentrations of bromoform were 6300 ng/L and 22,500 ng/L in source and finished water samples, respectively. Similar to finished water samples collected from the other networks, PFBA was the most frequently detected (50%) PFAS and was observed at the greatest concentration (9.44 ng/L). With some exceptions, PFAS were generally detected in a similar number of samples and at similar concentrations in source and finished water samples. Most of the detected pharmaceuticals were detected in one finished water sample, except for carbamazepine and sulfamethizole which were both detected in two samples.

### 3.2. Comparison of Contaminants in Source Waters

Differences between source waters among networks were observed for some contaminants. Although total pesticide concentrations were similar between GW-AG and surface water sources (*p* > 0.05), the number of pesticides detected in surface water source samples was significantly greater than groundwater (*p* < 0.001; Table S8, Figure S6). Surface water source samples also had significantly more wastewater indicators (*p* < 0.001) detected, higher total wastewater indicator concentrations (*p* < 0.01), and more pharmaceuticals detected (*p* < 0.05) when compared to groundwater (GW-WW) source samples (Table S8, Figure S7). Although others have reported differences in PFAS presence and/or magnitude in groundwater versus surface waters [42,43], results from our study did not reflect that pattern (*p* > 0.05; Table S8). This difference could be attributable to historical PFAS use in study regions, the specific groundwater source sampled, and/or aquifer properties, among other factors. Additionally, site selection for the current study was biased towards facilities at which contaminant presence was more likely. No significant differences in the number of contaminants detected or total sample concentrations in source waters were observed between GW-AG and GW-WW facilities (*p* > 0.05; Figure S8).

For groundwater source waters, those classified as vulnerable had significantly more and higher total sample concentrations for both pesticides and PFAS (*p* < 0.05; Figure 3, Table S8, Figures S9 and S10). One explanation for this difference is that vulnerability considers groundwater age (e.g., pre-modern, modern). Source waters designated as vulnerable are likely to be more influenced by activities on the landscape because of faster recharge rates and consequently may be more susceptible to contamination. This is consistent with Bexfield et al. [53] who reported higher detections frequencies in shallower unconfined wells. There was no significant difference (*p* > 0.05; Table S8) in the number of pharmaceuticals detected or total sample concentrations, likely because of few detections overall. Despite these differences, similar contaminants were detected in samples collected from both types of wells (e.g., several pesticide degradates, PFOSA). Although facilities located in vulnerable and non-vulnerable areas had similar types of point sources within the DWSMA, there tended to be fewer point sources associated with non-vulnerable facilities.

### 3.3. Human-Health Screening

Results of the human-health risk screening focus on finished water samples since these samples more closely reflect potential exposure. However, results for both source and finished water samples are provided in Tables S9 and S10. Bioactivity information for 73 (65%) of the detected contaminants in finished water samples was available in the ToxCast database. Guidance values were available for 68 (60%) of the detected contaminants, 23 of which were not represented in the ToxCast database. Considering the number of contaminants that could be screened, relatively few were detected at concentrations in finished water samples resulting in elevated concentrations (above 0.001 and 0.1 thresholds for EAR and TQ, respectively). However, testosterone, pentachlorophenol, PFOS, and PFOA did exceed screening values in at least one sample (Tables S9 and S10).

#### 3.3.1. Single Chemical Human-Health Risk Screening

**Figure 4:**
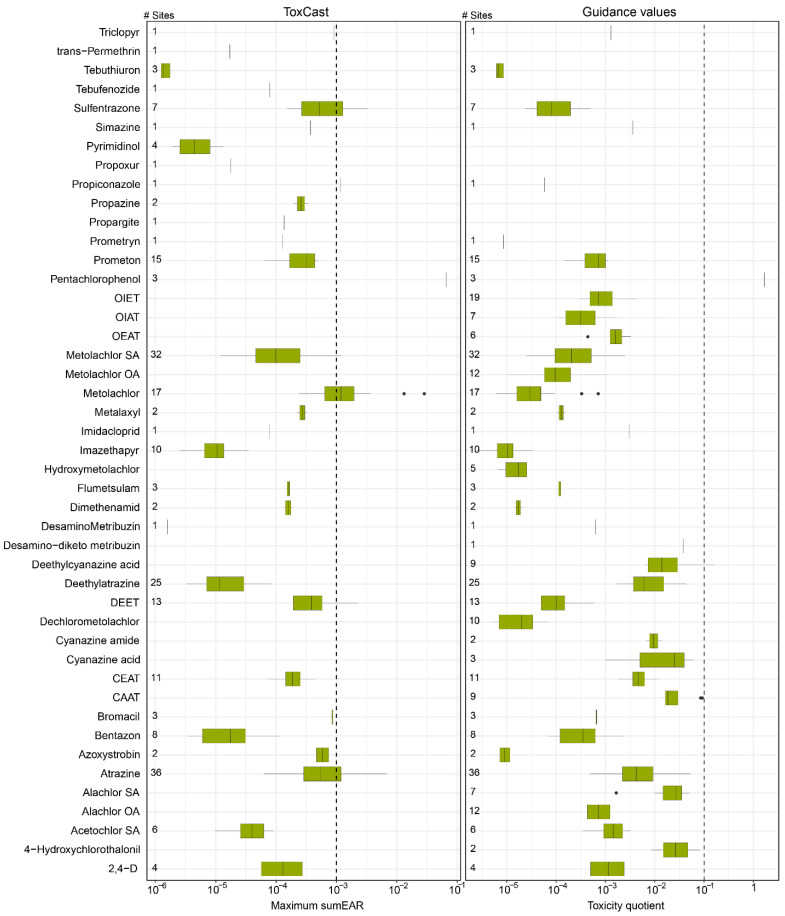
Boxplot summaries of health-risk screening for pesticide concentrations detected in finished drinking water samples collected in Minnesota, 2019–2022. The summation of maximum exposure-activity ratios (∑EAR) was determined by dividing environmental concentrations by activity concentrations from the U.S. Environmental Protection Agency’s ToxCast database. Toxicity quotients (TQ) were calculated by dividing environmental concentrations by guidance values from the Minnesota Department of Health (Table S2). The number of sites at which the contaminant was detected is displayed on the left edge of the graphs. The 25th, 50th, and 75th percentiles are represented by the bottom of the box, black line within the box, and top of the box, respectively. Whiskers extend to the minimum and maximum values and outliers are represented by individual dots. Only pesticides with at least one EAR > 0.001 or TQ > 0.1 are shown.

Compared to screening values, contaminant concentrations were relatively low. For 11 of the 18 pesticides with at least one EAR > 0.001 in source water samples, no EAR values in finished water samples were > 0.001. Of the pesticides with at least one exceedance, EAR or TQ exceeded the screening threshold by up to three times (Figure 4). Metolachlor and atrazine were exceptions, with 10 and 11 EAR exceedances, respectively. Metolachlor, which was included in USEPA Unregulated Contaminant Monitoring Rule, was identified as a herbicide with moderate toxic potential (in relation to 10 select nuclear receptors) [56]. Atrazine (along with alachlor SA) was identified as a priority contaminant from a risk ranking and prioritization analysis of contaminants in U.S. drinking water [6]. Using both screening methods, pentachlorophenol was the only pesticide to exceed a guidance value in finished water samples, at three different facilities. Furthermore, although several pesticides (atrazine, deethylatrazine, hydroxyatrazine, deethylcyanazine acid, metolachlor, metolachlor SA, DEET, and acetochlor OA) were detected in ≥20% of finished water samples, the potential risk to exposure of these contaminants individually is estimated to be low.

No pharmaceuticals exceeded available guidance values. However, one instance each of colchicine and thiabendazole was associated with an elevated concentration (Figure S11), the lowest percent of all the contaminant groups. Using both screening methods, concentrations were generally at least an order of magnitude below screening thresholds. Similar results of low potential risk of exposure to individual pharmaceuticals in drinking water have been reported in the U.S. [57] and Germany [58]. This low potential risk can be attributed to low occurrence and low (<50 ng/L) concentrations of pharmaceuticals in drinking waters [22,48,59] and efficient removal by drinking water processes [60].

Similar to other contaminant groups, wastewater indicators infrequently exceeded screening thresholds (Figure S12). One exception was bromoform (disinfection byproduct) for which 80% of detected concentrations resulted in EAR > 0.001 and 50% resulted in TQ > 0.1. Furthermore, bromoform exceeded the guidance value in finished water samples collected from two of the facilities at which it was measured. Bromoform is a byproduct of disinfection, which is necessary to remove harmful pathogens from drinking water. Disinfection byproducts (including bromoform) were reported as a contaminant group frequently exceeding health reference levels across the U.S. [6]. A couple of phosphate-based flame retardants [tri(2-butoxyethyl)phosphate and tris(dichloroisopropyl)phosphate] and bis(2-ethylhexyl) phthalate each resulted in one to three EAR values > 0.001. However, when compared against guidance values, only bis(2-ethylhexly) phthalate resulted in TQ > 0.1 (Table S10). Similar results for phosphate-based flame retardants have been observed in the U.S. and China [61,62].

State-based guidance values were available for six PFAS. Although PFAS were detected in finished water samples at up to 37 sites, most concentrations were < 10% of their respective guidance values. The USEPA has established maximum contaminants levels for five PFAS [PFOA (4 ng/L), PFOS (4 ng/L), PFHxS (10 ng/L), PFNA (10 ng/L), and HFPO-DA (10 ng/L)]; however, the MDH updated guidance values for PFOA (0.0079 ng/L) and PFOS (2.3 ng/L) in 2024 (Table S2; MDH, 2024). According to these updated values, 19 (100% of detections) and 4 (12%) finished water sample concentrations were above guidance values for PFOA and PFOS, respectively. Furthermore, all PFOA and PFOS concentrations resulted in TQ > 0.1 (Figure S13). PFBA was the most frequently detected PFAS in all water samples (41%) and finished water samples (44%) but was consistently found at concentrations two to three orders of magnitude lower than its guidance value. However, the PFBA guidance value is an order of magnitude greater than the next highest value. PFOSA was detected more frequently in finished water samples, compared to source, but no guidance value was available. The presence and magnitude of PFOSA in finished drinking water observed in our study generally agrees with observations from Canada [63] and Spain [64].

#### 3.3.2. Chemical Mixture Human-Health Screening

**Figure 5:**
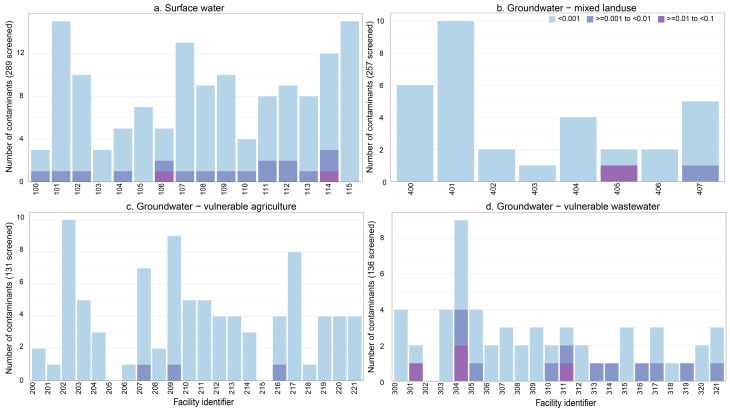
Stacked bar charts showing the number of contaminants with exposure-activity ratios (EAR) that fall within specified ranges (see legend) in finished water samples collected from (**a**) surface water sourced drinking facilities, (**b**) groundwater sourced drinking facilities located in areas of mixed agriculture and wastewater land use, (**c**) groundwater sourced drinking facilities located in agricultural and vulnerable areas, and (**d**) groundwater sourced drinking facilities located in areas with substantial wastewater sources and in vulnerable areas located within Minnesota, USA.

By assuming simple additivity, we can assess potential risks of exposure to contaminant mixtures. However, this analysis is constrained by the contaminants targeted in the study and limited to the 100 (of up to 513) contaminants that were detected. Generally, in each sample, ∑EAR_max_ values were lower in finished water samples, compared to source, and <0.001 (Figure S14). Additionally, when considering the vulnerability of a well to contamination, low potential risk was predicted at non-vulnerable wells. In fact, no contaminants were detected at an elevated concentration in any sample collected from non-vulnerable wells and sample ∑EAR_max_ values were low (<0.001; Figures S14 and S15). High ∑EAR_max_ values (>0.001) in individual samples were often driven by a few contaminants that were detected at relatively high concentrations. At SW and GW-WW facilities, this was often driven by bromoform. However, GW-AG facilities exhibited a different pattern in which several pesticides were detected at relatively low concentrations, but together resulted in a high sample ∑EAR_max_. Finished water samples collected from surface water facilities had elevated concentrations for up to three contaminants which resulted in ∑EAR_max_ > 0.001 (Figure 5). Finished water samples that had more than one contaminant present at elevated concentrations were most often associated with bromoform and metolachlor (Table S9). With one exception, one or two contaminants were detected at elevated concentrations in finished samples collected from vulnerable GW-WW facilities. Four contaminants, mostly wastewater indicators, were detected at elevated concentrations in the finished water sample collected from one facility (304; Figure 5; Table S9). Potential risk from exposure to contaminant mixtures, especially mixtures that include multiple types of contaminants, in drinking water is largely unknown because of uncertainties associated with chemical interactions and the implications on human health. Health risk from exposure to pharmaceutical mixtures in Dutch drinking water [65] and to mixtures of 14 various organic contaminants in Milanese drinking water [22] were determined to be low. Conversely, a previous study restricted to the greater Minneapolis-St. Paul area of Minnesota [20] and a study conducted throughout the U.S. [66] observed elevated ∑EAR_max_ (>0.001) in 79% and 95% of finished water samples from community water supply facilities, which were partially attributed to volatile organic compounds not analyzed in this current study.

## 4. Conclusions

There are many data gaps related to the distribution of unregulated contaminants in finished drinking water. Our study indicates that several of the contaminants included in the study are present in Minnesota finished drinking water; at least one contaminant was detected in finished water collected from 90% of sampled facilities. Of the 513 contaminants analyzed, 117 were detected in finished water samples at least one time, with concentrations ranging from <1 to 2500 ng/L. However, contaminants were generally detected at low frequencies and/or low concentrations. Overall, commonly used pesticides and/or their degradates (e.g., atrazine, metolachlor) were the most frequently detected contaminants. Conversely, most pharmaceuticals were detected in relatively few (<5) samples, despite some of the source waters being located in areas that may be impacted by discharges such as wastewater effluents or subsurface treatment system effluents. We detected fewer contaminants (e.g., PFAS, pesticides) in non-vulnerable wells, compared to vulnerable, indicating that characteristics describing the vulnerability of a drinking water well to contamination are important indicators for the presence of contaminants. This information can be used to guide site selection for monitoring and efficient use of resources.

Our health-based risk screening indicates a relatively low risk to consumers, with concentrations generally at least an order of magnitude lower than available screening values. Furthermore, our screening indicated few (<5) contaminants of potential concern within individual finished water samples. However, since guidance values do not exist for many unregulated contaminants, our screening likely underestimates overall risk. Furthermore, little is understood about the effects of exposure to contaminant mixtures. Our assessment also did not include regulated contaminants, which may contribute to the potential risk of exposure to mixtures. Data from our study could be used to identify typical mixtures (including composition and concentrations) of unregulated contaminants that may reflect the type of source water and anthropogenic activities on the landscape. This information could be used to inform toxicological studies focused on expected mixtures to provide information related to potential health risks.

Results from this study were (and may be in the future) used to enhance drinking water protection activities at MDH. For example, several contaminants that were detected relatively frequently and at high concentrations were nominated for development of health-based guidance values. Additionally, this study informed development of a framework to guide Minnesota public water facility response to detections of unregulated contaminants, including follow up monitoring and public notifications. In one instance, a system-level change was implemented to minimize and/or remove potential public health risk to exposure of unregulated contaminants. Results from this study can also be used to inform future targeted monitoring based on well vulnerability, water source, and land use within the watershed or DWSMA. Additionally, our results can be used to prioritize contaminants for future monitoring for efficient use of resources. For example, our risk screening indicates that pesticides may be a higher priority compared to pharmaceuticals, based on number of detections and concentrations. Continued monitoring of unregulated contaminants at Minnesota’s drinking water facilities (and elsewhere) may be useful for improving our understanding of long-term hazards associated with public exposure.

## Figures and Tables

**Table 1:** Contaminants analyzed in samples collected from each Minnesota community drinking water facility network. Table S2 contains additional details regarding specific contaminants analyzed within each group. ‘Groundwater agriculture’ refers to groundwater sourced facilities likely to be influenced by agricultural activities on the landscape. ‘Groundwater wastewater’ refers to groundwater sourced facilities likely to be influenced by wastewater inputs to the environment. ‘Groundwater agriculture + wastewater’ refers to groundwater facilities likely to be influenced by both agricultural activities on the landscape and wastewater inputs to the environment.

Contaminant Group	Number of Contaminants Analyzed ^a^	Surface Water (SW)	Groundwater Agriculture (GW-AG)	Groundwater Wastewater (GW-WW)	Groundwater Agriculture + Wastewater (GW-AG/WW)
Wastewater indicators ^b^	49	x		x	x
Pharmaceuticals and personal care products	165	x		x ^c^	x
Per- and polyfluoroalkyl substances (PFAS)	40	x	x	x	x
Benzotriazoles/ benzothiazoles	10	x		x ^d^	
Illicit drugs	4	x			
Alkylphenols and alkylphenol ethoxylates	5	x			
Hormones ^e^	16	x			
Pesticides and pesticide degradates	224	x	x ^f^		x ^f^
Total number of contaminants	513	513	256	272	480

^a^ Some networks included a subset of the contaminants within a group. The number of contaminants analyzed represents the total number of contaminants analyzed across all sampling networks. ^b^ Wastewater indicators is a diverse contaminant group including flame retardant, flavor and fragrance, industrial, lifestyle, multi-use, polycyclic aromatic hydrocarbon (PAH), plant sterol, plastic additive, sterol contaminants, and bromoform (disinfection byproduct). ^c^ A subset of pharmaceuticals (*n* = 59) and personal care products were analyzed in non-vulnerable wells. ^d^ Benzotriazoles were analyzed only in samples collected from non-vulnerable wells. ^e^ Excludes the hormone mestranol. ^f^ Nine additional pesticides including four atrazine and five cyanazine degradates were analyzed.

## Data Availability

Data used in analysis for this study are available in a U.S. Geological Survey data release: https://doi.org/10.5066/P14VWFHB.

## Supplementary Materials

The following supporting information can be downloaded at: https://www.mdpi.com/article/10.3390/ijerph22070976/s1. Detailed analytical methods. Figure S1: Violin plots of total sample concentrations and number of detections, by contaminant group, in source and finished water samples collected from surface water-sourced community water facilities, Minnesota, 2019–2022.; Figure S2: Violin plots of total sample concentrations and number of detections, by contaminant group, in source and finished water samples collected from groundwater sourced community water systems classified as vulnerable and located within agriculture areas, Minnesota, 2019–2022.; Figure S3: Violin plots of total sample concentrations and number of detections, by contaminant group, in source and finished water samples collected from groundwater sourced community water systems classified as non-vulnerable and located within agriculture areas, Minnesota, 2019–2022.; Figure S4: Violin plots of total sample concentrations and number of detections, by contaminant group, in source and finished water samples collected from groundwater sourced community water systems classified as vulnerable and located within developed areas, Minnesota, 2019–2022.; Figure S5: Violin plots of total sample concentrations and number of detections, by contaminant group, in source and finished water samples collected from groundwater sourced community water systems classified as non-vulnerable and located within developed areas, Minnesota, 2019–2022.; Figure S6: Violin plots of total sample concentrations and number of detections, by contaminant group, in source water samples collected from groundwater sourced community water facilities located within agricultural areas and surface water-sourced community water facilities, Minnesota, 2019–2022.; Figure S7: Violin plots of total sample concentrations and number of detections, by contaminant group, in source water samples collected from groundwater sourced community water facilities located within developed areas (wastewater) and surface water-sourced community water facilities, Minnesota, 2019–2022.; Figure S8: Violin plots of total sample concentrations and number of detections, by contaminant group) in source water samples collected from groundwater sourced community water facilities located within agriculture and developed areas (wastewater, Minnesota, 2019–2022.; Figure S9: Violin plots of total sample concentrations and number of detections, by contaminant group, in source water samples collected from groundwater sourced community water facilities located within developed areas classified as vulnerable and non vulnerable, Minnesota, 2019–2022.; Figure S10: Violin plots of total sample concentrations and number of detections, by contaminant group, in source water samples collected from groundwater sourced community water facilities located within agriculture areas classified as vulnerable and non vulnerable, Minnesota, 2019–2022.; Figure S11: Boxplot summaries of health-risk screening for pharmaceutical concentrations detected in finished drinking water samples collected in Minnesota, 2019–2022.; Figure S12: Boxplot summaries of health-risk screening for wastewater indicator concentrations detected in finished drinking water samples collected in Minnesota, 2019–2022.; Figure S13: Boxplot summaries of health-risk screening for per- and polyfluoroalkyl concentrations detected in finished drinking water samples collected in Minnesota, 2019–2022.; Figure S14: Bar chart comparing sum of maximum exposure-activity ratios (EAR) for individual source and finished water samples collected at individual facilities in Minnesota, 2019–2022.; Figure S15: Bar charts showing the number of contaminants with exposure-activity ratios (EAR) that fall within specified ranges (see legend) in source and finished water samples collected from (a) non vulnerable groundwater sourced drinking facilities located in developed areas (wastewater), and (b) non vulnerable groundwater sourced drinking facilities located in agricultural areas.; Table S1: Drinking water facilities at which source and finished water samples were collected throughout Minnesota, 2019–2022.; Table S2: Contaminants analyzed in source and finished drinking waters collected throughout Minnesota, 2019–2022 and associated guidance values used to screen environmental data.; Table S3: Summary of contaminant surrogates analyzed in source and finished drinking waters collected throughout Minnesota, 2019–2022, including networks sampled, number of analyses (N), minimum percent recovery, mean percent recovery, median percent recovery, median percent recovery, maximum percent recovery, and the standard deviation.; Table S4: Concentrations of contaminants detected in field or trip blank samples, 2019–2022.; Table S5: Comparison of concentrations detected in environmental samples and associated replicate samples.; Table S6: Summary of contaminant surrogates analyzed in replicate samples from source and finished drinking waters collected throughout Minnesota, 2019–2022, including networks sampled, number of analyses (N), minimum percent recovery, mean percent recovery, median percent recovery, median percent recovery, maximum percent recovery, and the standard deviation.; Table S7: Summary of contaminants by network and sample type (source water, finished water), including sample size (N), number of times detected, minimum concentration, and maximum concentration.; Table S8: Results of Wilcoxon signed-rank sum test with Pratt adjustment for comparisons of paired source and finished drinking water samples and PERMANOVA test for comparisons of source waters.; Table S9: Summary of exposure-activity ratios (EARs) determined by comparing environmental concentrations against bioactivity data available in the U.S. Environmental Protection Agency’s ToxCast database.; Table S10: Summary of toxicity quotients determined by comparing environmental concentrations against guidance and screening values available from the Minnesota Department of Health.
