# Supplementary material for: Emerging Contaminants in Source and Finished Drinking Waters Across Minnesota (U.S.) and Potential Health Implications
Source: Int J Environ Res Public Health. 2025 Jun 20;22(7):976. doi: 10.3390/ijerph22070976 (PMC12294311; doi:10.3390/ijerph22070976)
Supplement: Supplementary file 1 [file ijerph-22-00976-s001.zip › ijerph-3633479-supplementary/ijerph-3633479_SI_R1_clean.pdf]

Supplemental information for "Emerging contaminants in source and finished drinking waters across Minnesota (U.S.) and potential health implications"

Sarah M. Elliott[a], Aliesha L. Krall[a], Jane R. de Lambert[b], Maya D. Gilchrist[b], Stephen W. Robertson[b]

[a]U.S. Geological Survey, 2280 Woodale Drive, Mounds View, Minnesota, 55112, USA
[b]Minnesota Department of Health, 625 Robert Street North, Saint Paul, Minnesota, 55155, USA

Contents

Analytical methods 2
  Benzotriazoles 2
  Illicit drugs 2
  Alkylphenols 2
  Per- and polyfluoroalkyl substances (PFAS) 3
  Pharmaceuticals and hormones 3
Disclaimer 3
Figures 4

Analytical methods

Details regarding analytical methods for those methods that are not publicly available are provided. For chemical specific information (e.g., Chemical Abstracts Registry Number) refer to Table S2.

Benzotriazoles

Whole water samples were analyzed for eight benzotriazole and benzothiazoles (referred to hereafter as 'benzotriazoles') by the Minnesota Department of Health Public Health Laboratory (St. Paul, Minnesota, U.S.). Upon receipt at the laboratory, samples were stored frozen at <-10°C until analysis. Samples were thawed in a water bath, if necessary, centrifuged to remove suspended particulates, and an internal standard was added. Then, 900 µL of the sample was analyzed by high-performance liquid chromatography (HPLC) tandem mass spectrometry (MS/MS). For each batch of 20 samples, a method blank, laboratory control spike, matrix spike, and sample duplicate were analyzed for method verification. Additionally, a new calibration curve was run or a previous calibration curve was validated for each batch. Calibration curves consisted of at least five concentration levels, with one at or below the reporting level. Samples containing concentrations above the calibration range were diluted so that the concentration would fall within the calibration range and reanalyzed. Positive identification of the target analytes was based on comparison of the relative retention-time of a peak-of-interest to its internal standard, inclusion of two mass spectral transitions of the suspect analyte, and the ratio of the area of the two mass spectral transitions. Concentrations were then determined using a 1/x weighted linear or quadratic regression based on internal standards.

Illicit drugs

Whole water samples were analyzed for eight illicit drugs by the Minnesota Department of Health Public Health Laboratory (St. Paul, Minnesota, U.S.). Upon receipt at the laboratory, samples were stored frozen at <-10°C until analysis. After thawing samples in a water batch, a 1 mL aliquot was acidified with 1 µL of formic acid, and internal standard solution was added, and the aliquot was centrifuged. At least 750 µL of the supernatant was transferred to an autosampler vial and analyzed directly by large-volume injection HPLC-MS/MS with electrospray ionization operating in positive polarity. For each batch of 20 samples, a method blank, laboratory control spike, matrix spike, and sample duplicate were analyzed for method verification. Additionally, a new calibration curve was run or previous calibration curve was validated for each batch. Calibration curves consisted of at least five concentration levels, with one at or below the reporting level. Samples containing concentrations above the calibration range we diluted so that the concentration would fall within the calibration range and reanalyzed. Positive identification of the target analytes was based on comparison of the relative retention-time of a peak-of-interest to its internal standard, inclusion of two mass spectral transitions of the suspect analyte, and the ratio of the area of the two mass spectral transitions. Concentrations were then determined using a 1/x weighted linear or quadratic regression based on internal standards.

Alkylphenols

Whole water samples were analyzed for 4-nonylphenol, 4-nonylphenol monoethoxylate, 4-nonylphenol diethoxylate, and 4-n-octylphenol by SGS AXYS Analytical Services Ltd. (British Columbia, Canada). Upon receipt at the laboratory, ^{13}C-labelled surrogate standards were added to the environmental samples. Samples were extracted using aqueous acetylation and liquid-liquid extraction with hexane. The hexane extract was derivatized by non-aqueous acetylation and cleaned up by chromatography on a silica column. Approximately 500 µL of extract were analyzed on a capillary gas chromatography column

coupled to a low-resolution mass spectrometer operated in the electron ionization mode using multiple ion detection. Calibration curves consisted of five concentrations within the working range. Identification of the compounds was based on peak responses for the quantification ions, retention time, peak maxima for quantification and confirmation ions, and relative ion abundance ratio. Quantification was determined with respect to labelled surrogate standards.

Per- and polyfluoroalkyl substances (PFAS)

Whole water samples were analyzed for 40 PFAS compounds. Samples were spiked with isotopically labeled surrogate standards, then extracted and cleaned by solid phase extraction. Extracts were analyzed using an ultrahigh performance liquid chromatography coupled to a triple quadrupole mass spectrometer run at unit mass resolution in the multiple reaction monitoring mode using negative electrospray ionization. Quantification of identified compounds were determined by isotope dilution/internal standard quantification. Calibration curves consisted of five or more concentrations. Concentrations were quantified using the isotope dilution/internal standard method. Concentrations are determined as the total of linear and branched isomers.

Pharmaceuticals and hormones

Water samples were analyzed for 59 pharmaceuticals and 17 hormones. Sample pH was adjusted to 2 and spiked surrogates were added. The samples were then filtered and the filtrate was cleaned by solid phase extraction. Samples were analyzed on an ultra-performance liquid chromatograph coupled to a triple quadrupole mass spectrometer run in multiple reaction monitoring mode. Quantification of analytes was completed using the peak areas of the precursor ion/product ion transitions. Some analytes were analyzed in the electrospray ionization in the positive mode, and some were analyzed in the negative mode.

Disclaimer

Any use of trade, firm, or product names is for descriptive purposes only and does not imply endorsement by the U.S. Government

Figures

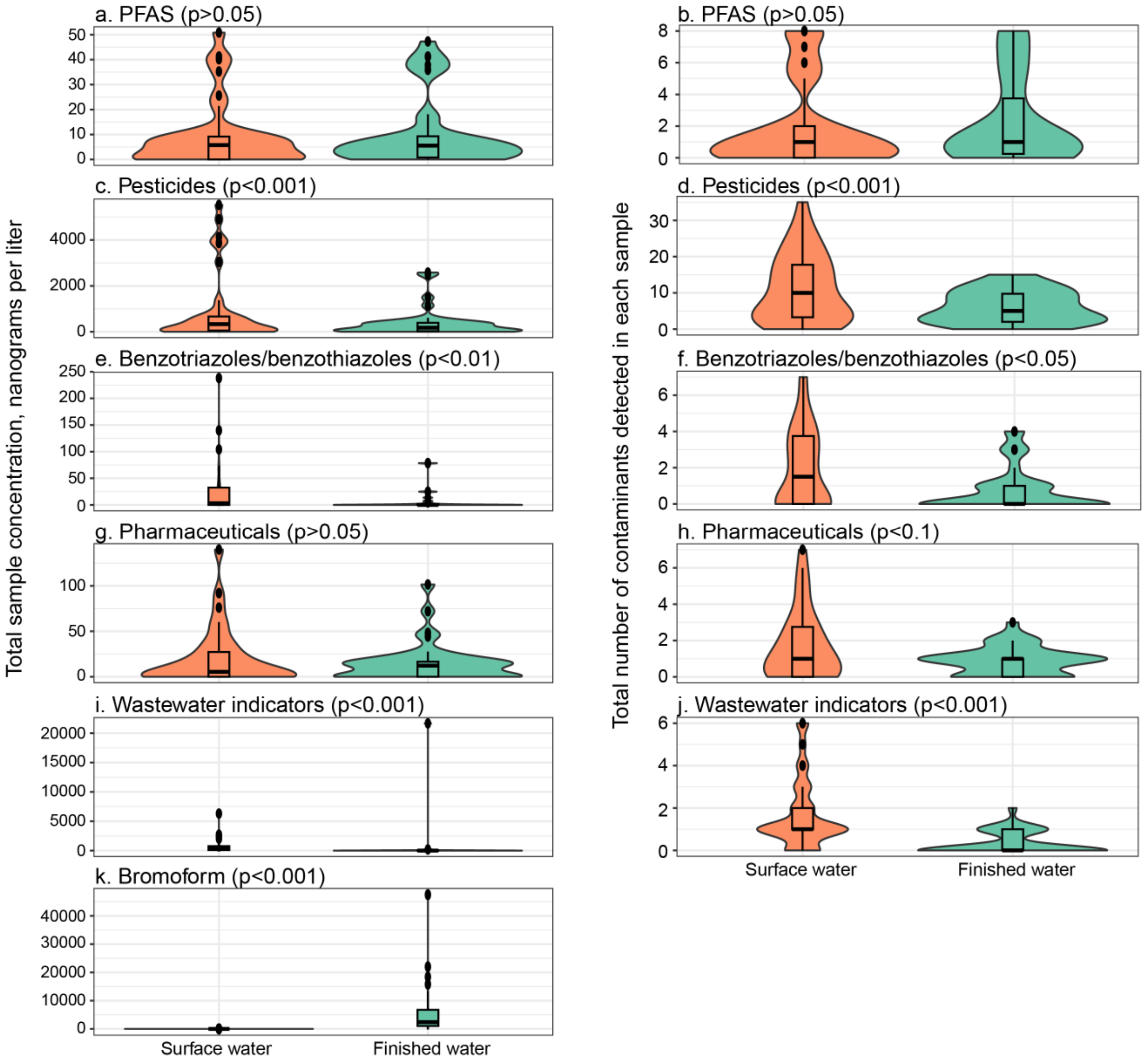

Figure S1. Violin plots of total sample concentrations and number of detections, by contaminant group, in source and finished water samples collected from surface water-sourced community water facilities, Minnesota, 2019-2022. The p-value assessing statistical significance between paired source and finished water samples is provided. PFAS, per- and polyfluoroalkyl substances

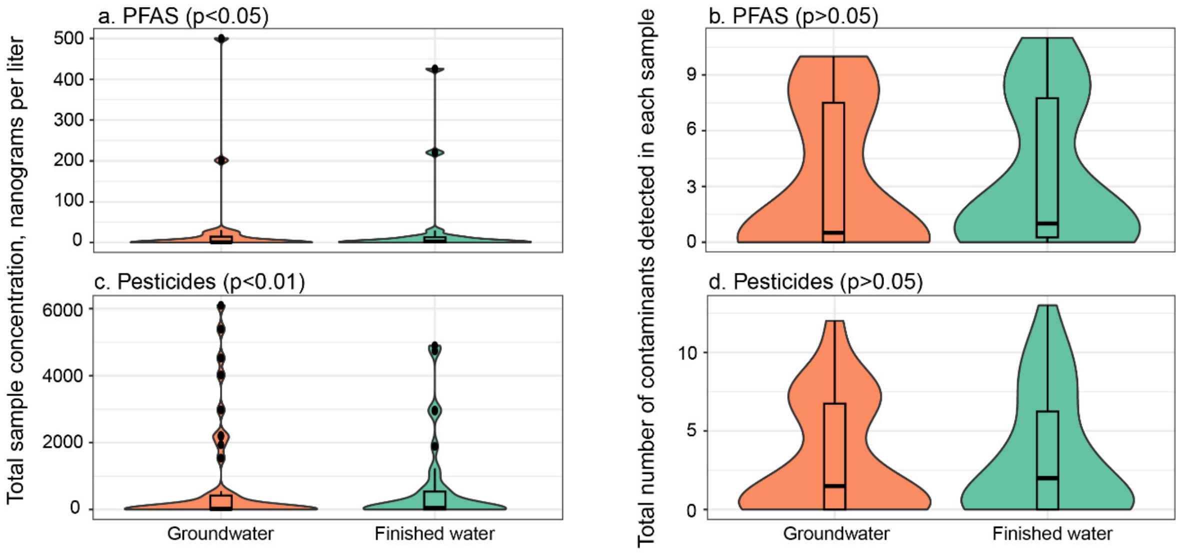

Figure S2. Violin plots of total sample concentrations and number of detections, by contaminant group, in source and finished water samples collected from vulnerable groundwater sourced public supply facilities likely to be influenced by agricultural activities on the landscape, Minnesota, 2019-2022. The p-value assessing community water statistical significance between paired source and finished water samples is provided. PFAS, per- and polyfluoroalkyl substances

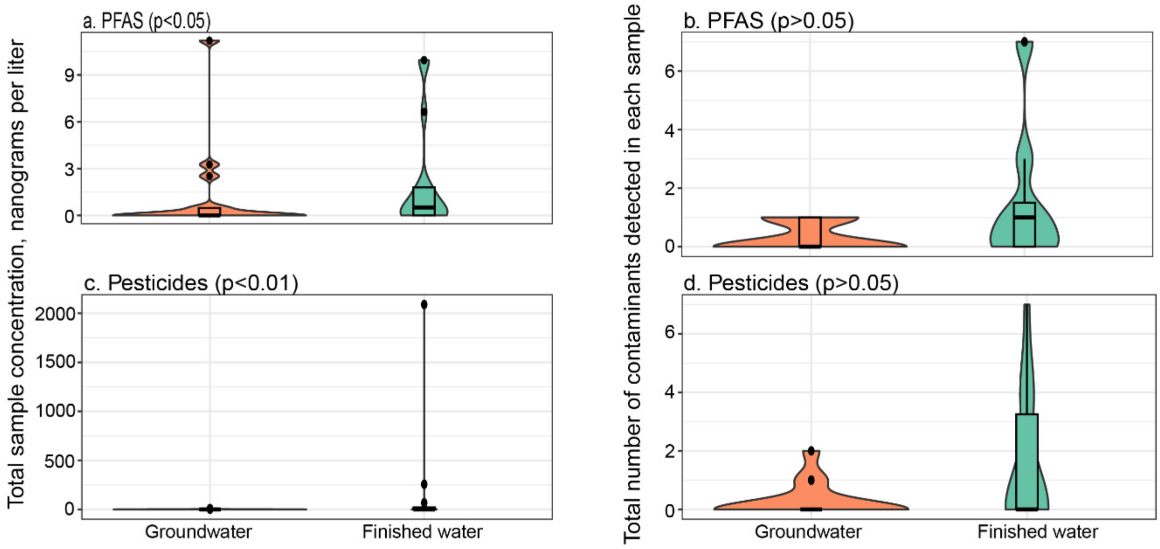

Figure S3. Violin plots of total sample concentrations and number of detections, by contaminant group, in source and finished water samples collected from non-vulnerable groundwater sourced community public water facilities likely to be influenced by agricultural activities on the landscape, Minnesota, 2019-2022. The p-value assessing statistical significance between paired source and finished water samples is provided. PFAS, per- and polyfluoroalkyl substances

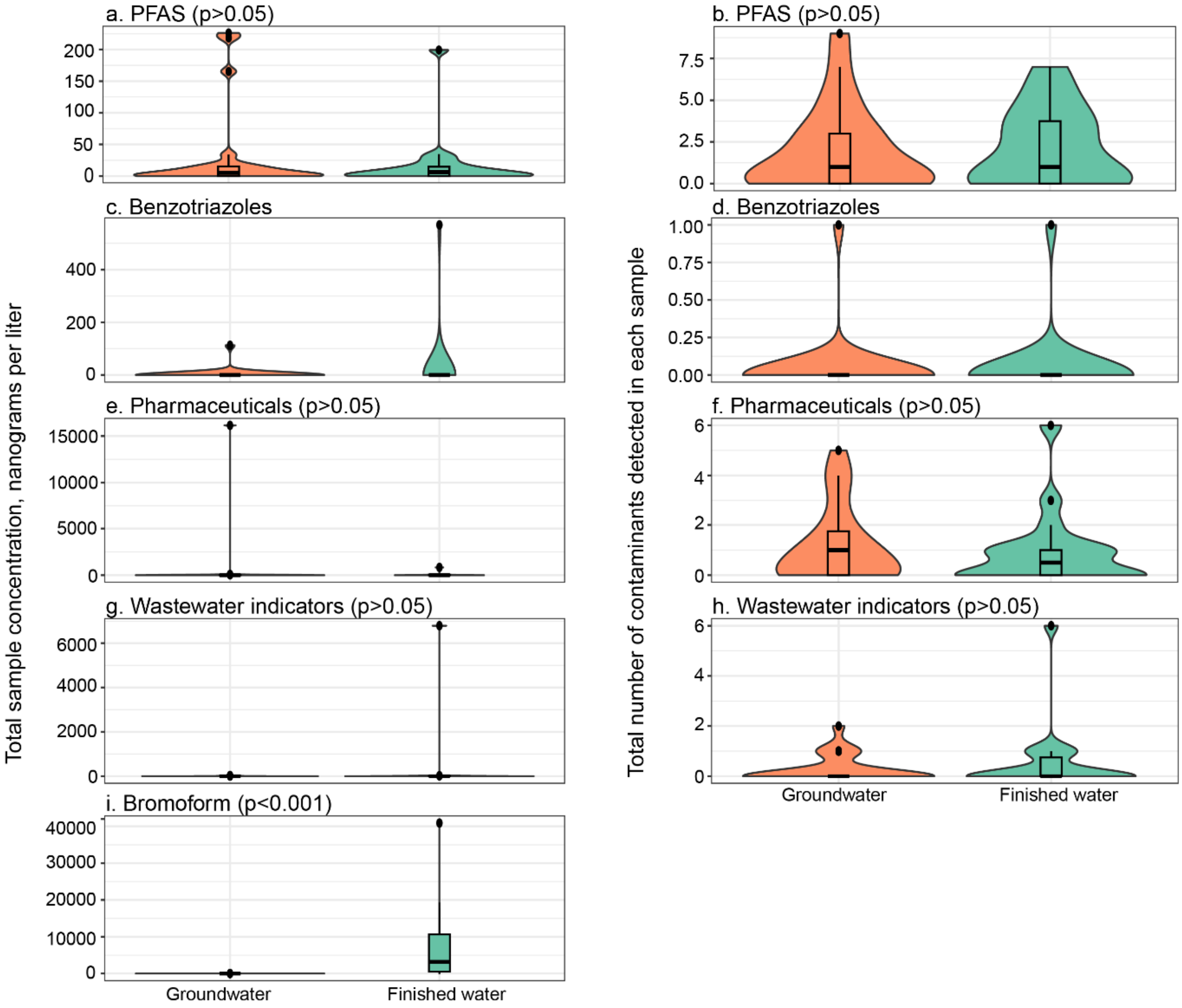

Figure S4. Violin plots of total sample concentrations and number of detections, by contaminant group, in source and finished water samples collected from vulnerable groundwater sourced community public facilities likely to be influenced by wastewater inputs to the environment, Minnesota, 2019-2022. The p-value assessing statistical significance between paired source and finished water samples is provided. No p-value indicates no statistical comparison. PFAS, per- and polyfluoroalkyl substances

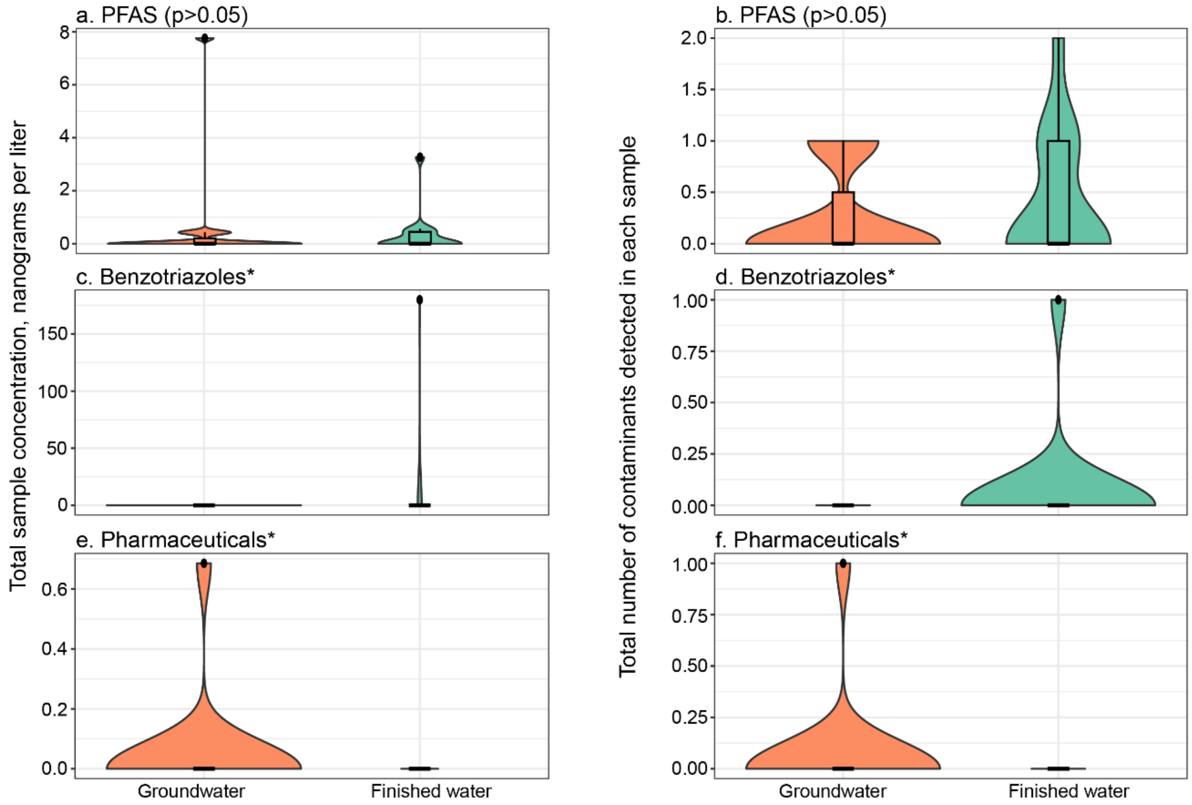

Figure S5. Violin plots of total sample concentrations and number of detections, by contaminant group, in source and finished water samples collected from non-vulnerable groundwater sourced community water supply facilities likely to be influenced by wastewater inputs to the environment, Minnesota, 2019-2022. The p-value assessing statistical significance between paired source and finished water samples is provided. No p-value indicates no statistical comparison. Asterisks indicate that there not even detections in samples to conduct statistical analyses. PFAS, per- and polyfluoroalkyl substances

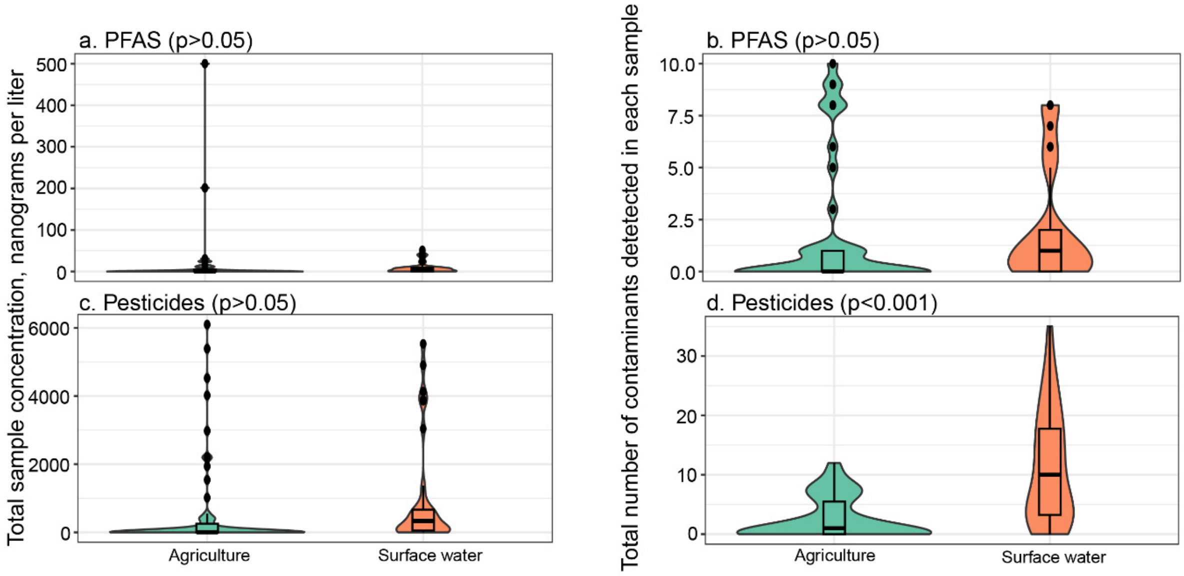

Figure S6. Violin plots of total sample concentrations and number of detections, by contaminant group, in source water samples collected from vulnerable groundwater sourced community water supply facilities likely to be influenced by agricultural activities on the landscape and surface water-sourced community water facilities, Minnesota, 2019-2022. The p-value assessing statistical significance between source water samples is provided. PFAS, per- and polyfluoroalkyl substances

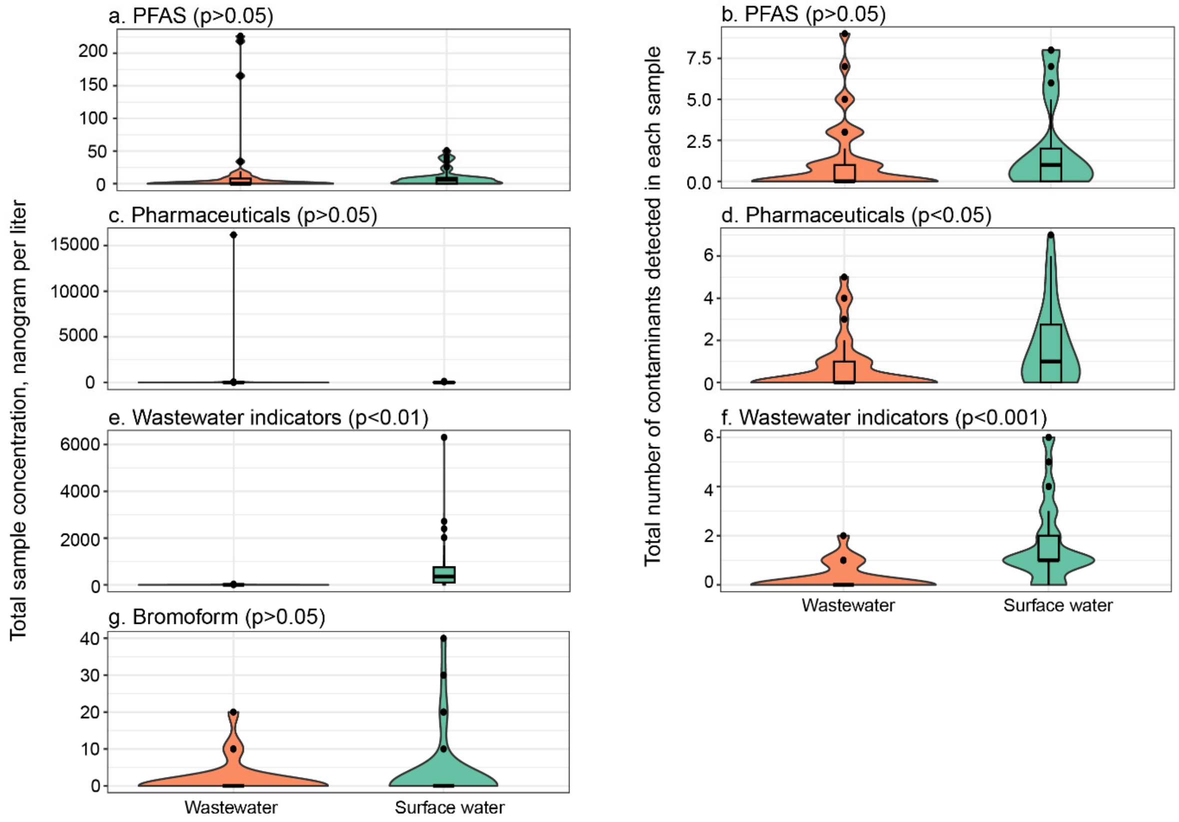

Figure S7. Violin plots of total sample concentrations and number of detections, by contaminant group, in source water samples collected from vulnerable groundwater sourced community water supply facilities likely to be influenced by wastewater inputs to the environment and surface water-sourced community water facilities, Minnesota, 2019-2022. The p-value assessing statistical significance between source water samples is provided. PFAS, per- and polyfluoroalkyl substances

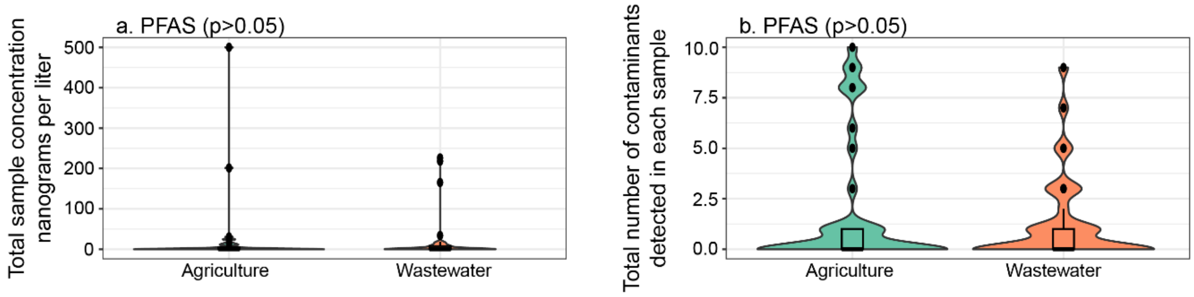

Figure S8. Violin plots of total sample concentrations and number of detections, by contaminant group) in source water samples collected from vulnerable groundwater sourced community water facilities likely to be influenced by agricultural activities on the landscape and likely to be influenced by wastewater inputs to the environment (Minnesota, 2019-2022. The p-value assessing statistical significance between source water samples is provided. PFAS, per- and polyfluoroalkyl substances

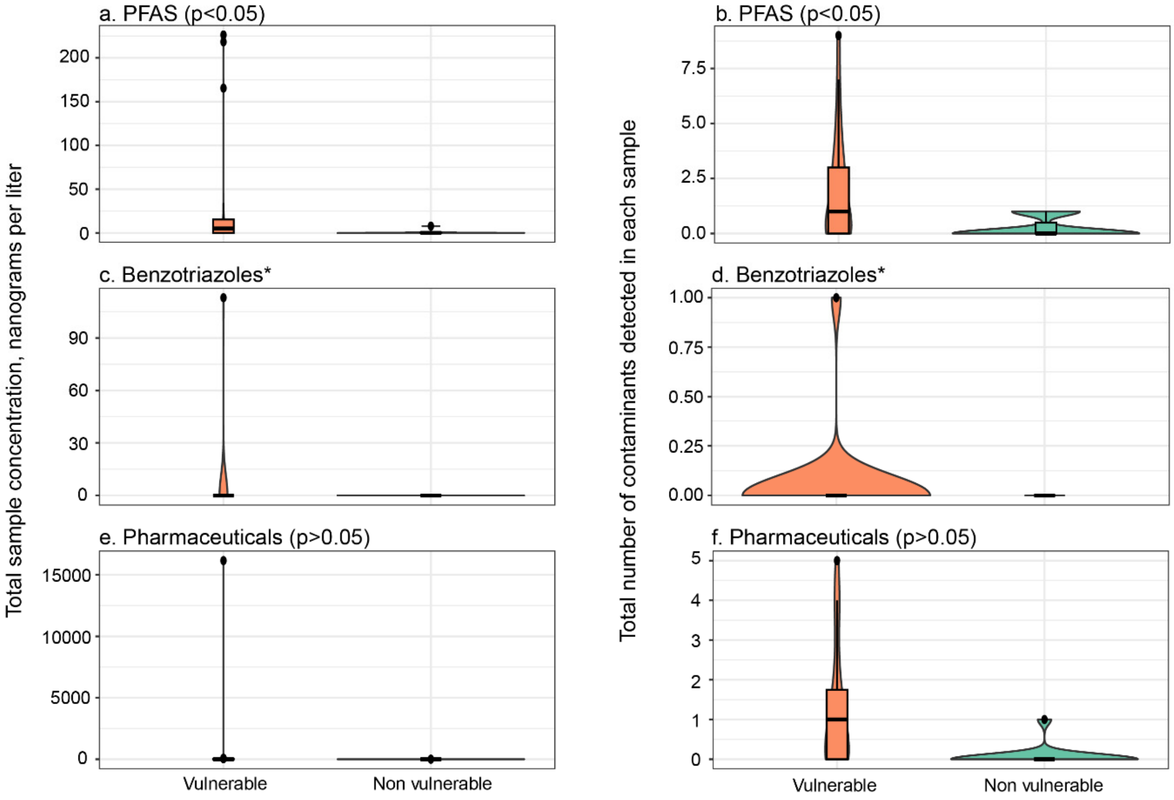

Figure S9. Violin plots of total sample concentrations and number of detections, by contaminant group, in source water samples collected from groundwater sourced community water supply facilities likely to be influenced by wastewater inputs to the environment classified as vulnerable and non vulnerable, Minnesota, 2019-2022. The p-value assessing statistical significance between source water samples is provided. Asterisks indicate that there not even detections in samples to conduct statistical analyses.

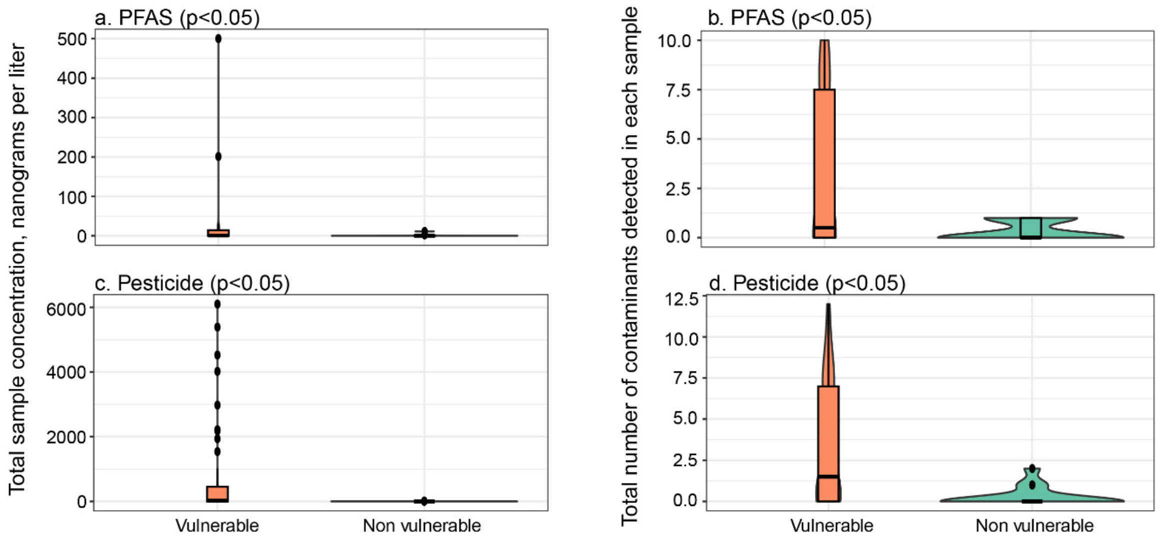

Figure S10. Violin plots of total sample concentrations and number of detections, by contaminant group, in source water samples collected from groundwater sourced community water supply facilities likely to be influenced by agricultural activities on the landscape classified as vulnerable and non vulnerable, Minnesota, 2019-2022. The p-value assessing statistical significance between source water samples is provided.

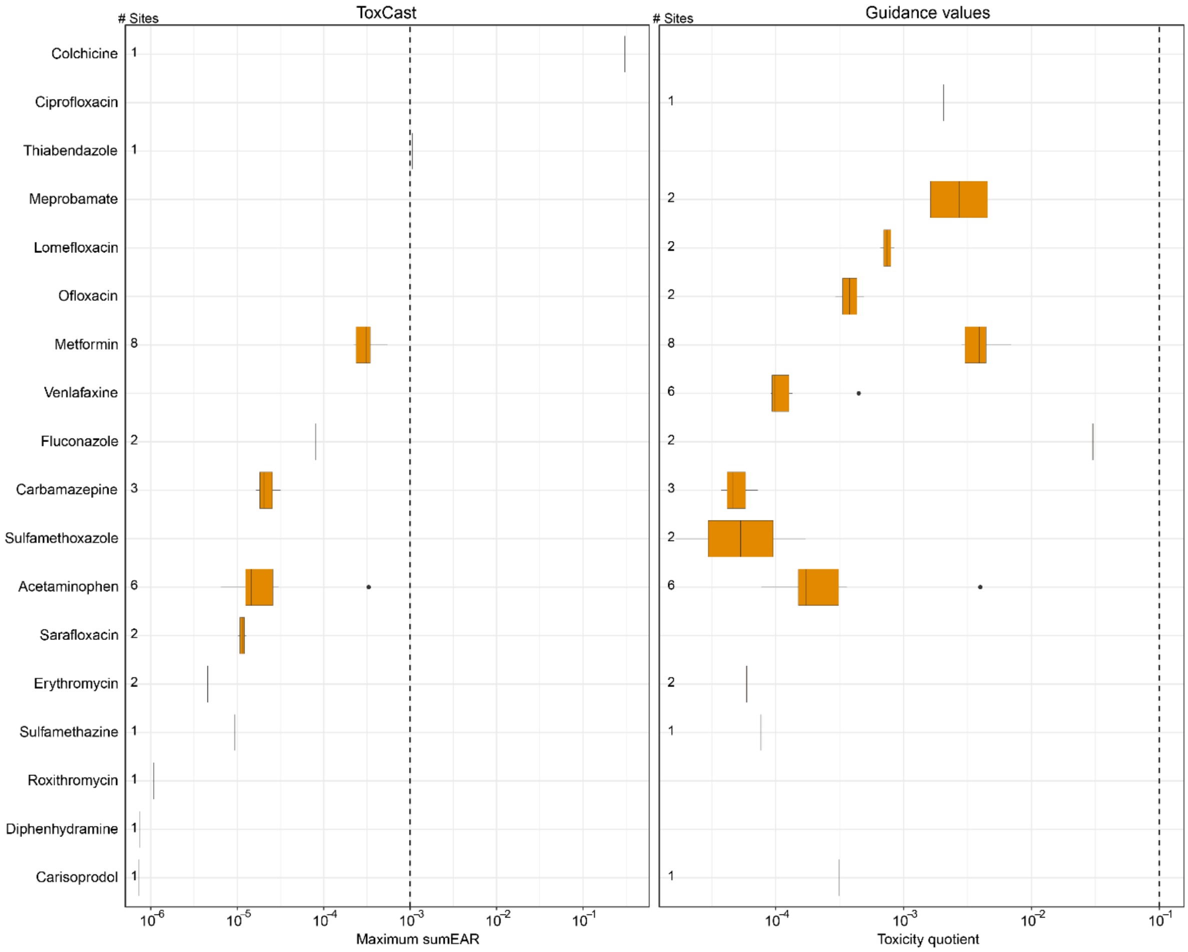

Figure S21. Boxplot summaries of health-risk screening for pharmaceutical concentrations detected in finished drinking water samples collected in Minnesota, 2019-2022. The maximum summation of exposure-activity ratios (sumEAR) was determined by dividing environmental concentrations by activity concentrations from the U.S. Environmental Protection Agency's ToxCast database. Toxicity quotients (TQ) were calculated by dividing environmental concentrations by available guidance values from the Minnesota Department of Health (Table S2). Number of sites at which the contaminant was detected are displayed on the left edge of the graphs. The 25th, 50th, and 75th percentiles are represented by the bottom of the box, black line within the box, and top of the box, respectively. Whiskers extend to the minimum and maximum values and outliers are represented by individual black dots. Only pharmaceuticals with at least one EAR >0.001 or TQ >0.1 are shown.

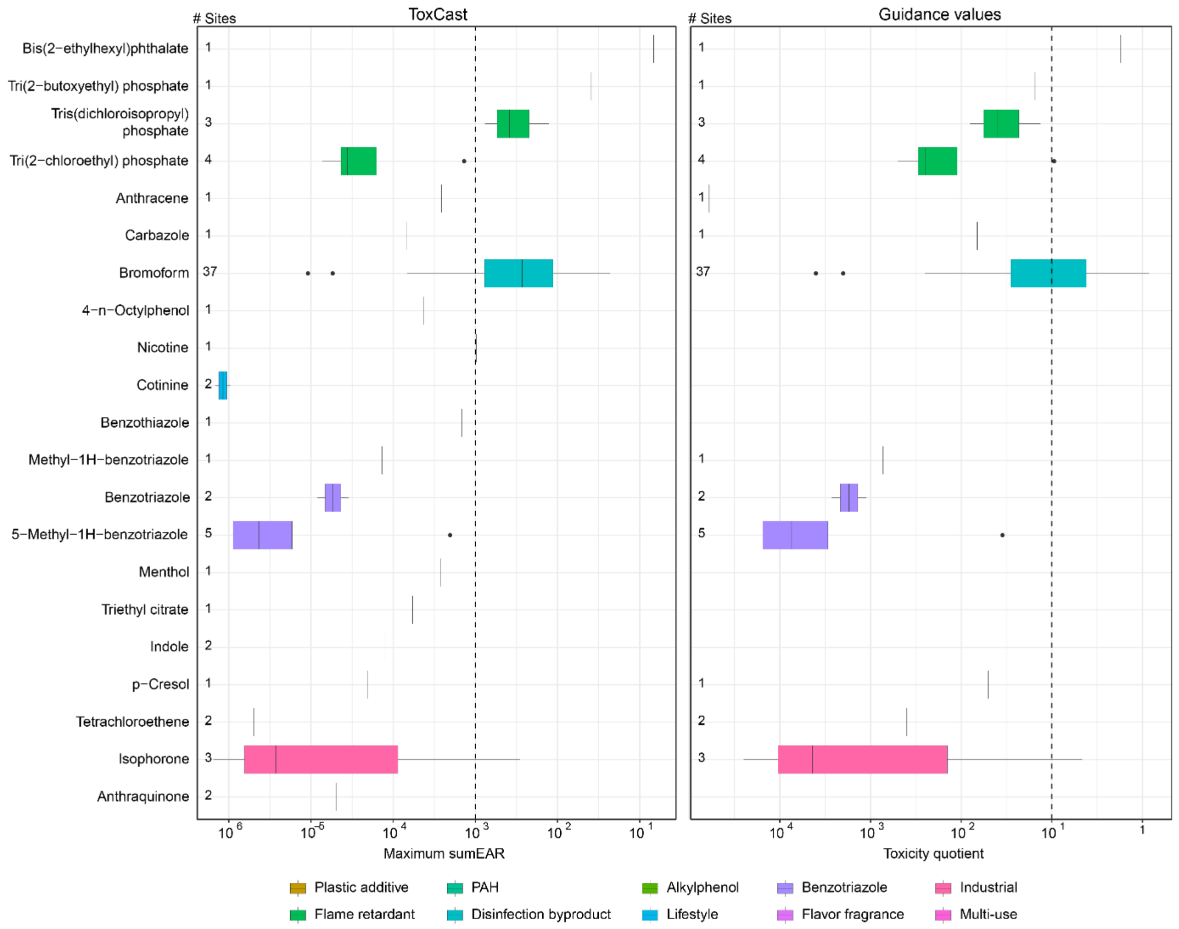

Figure S13. Boxplot summaries of health-risk screening for wastewater indicator concentrations detected in finished drinking water samples collected in Minnesota, 2019-2022. The maximum summation of exposure-activity ratios (sumEAR) was determined by dividing environmental concentrations by activity concentrations from the U.S. Environmental Protection Agency's ToxCast database. Toxicity quotients (TQ) were calculated by dividing environmental concentrations by available guidance values from the Minnesota Department of Health (Table S2). Number of sites at which the contaminant was detected are displayed on the left edge of the graphs. The 25th, 50th, and 75th percentiles are represented by the bottom of the box, black line within the box, and top of the box, respectively. Whiskers extend to the minimum and maximum values and outliers are represented by black individual dots. Only wastewater indicators with at least one EAR >0.001 or TQ >0.1 are shown. PAH, polycyclic aromatic hydrocarbon

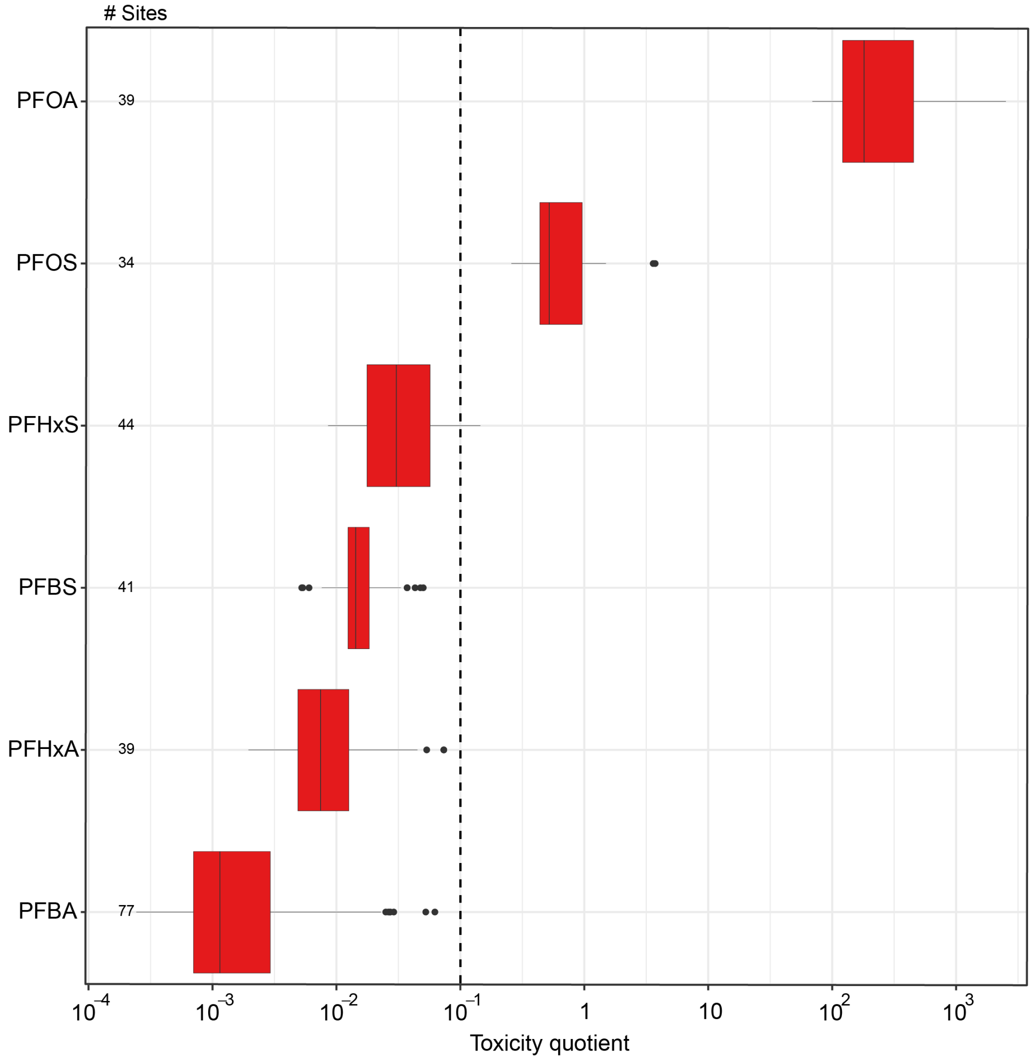

Figure S14. Boxplot summaries of health-risk screening for per- and polyfluoroalkyl concentrations detected in finished drinking water samples collected in Minnesota, 2019-2022. Toxicity quotients were calculated by dividing environmental concentrations by available guidance values from the Minnesota Department of Health (Table S2). Number of sites at which the contaminant was detected are displayed on the left edge of the graphs. The 25th, 50th, and 75th percentiles are represented by the bottom of the box, black line within the box, and top of the box, respectively. Whiskers extend to the minimum and maximum values and outliers are represented by individual black dots.

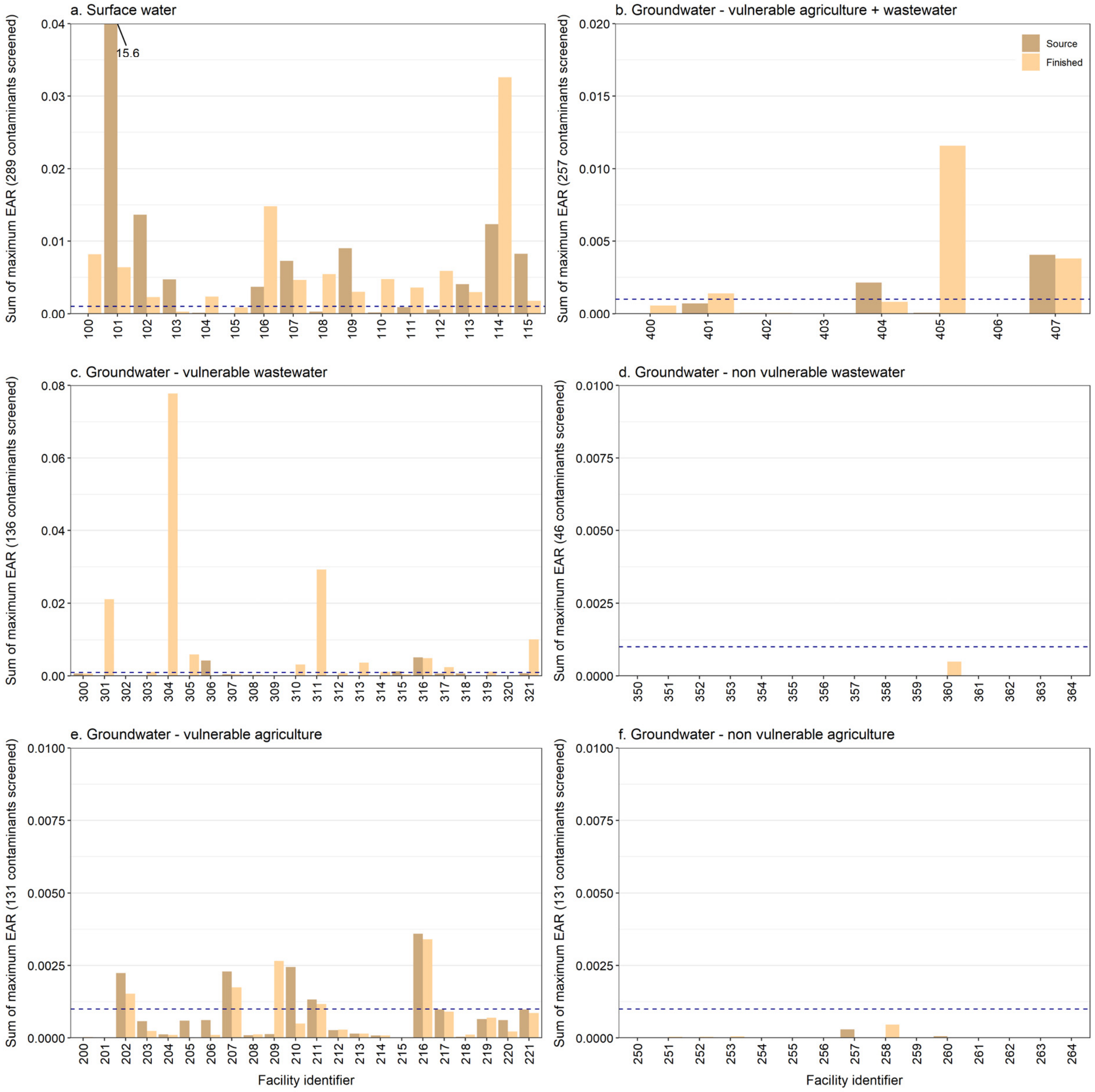

Figure S15. Bar chart comparing sum of maximum exposure-activity ratios (EAR) for individual source and finished water samples collected at individual facilities in Minnesota, 2019-2022. The sum of maximum EAR was calculated by determining summing the maximum EAR values for all chemicals detected in the sample. The dotted blue line indicates a screening threshold (0.001) to prioritize samples that may pose a greater risk for human health. If no bar is present, no contaminants were detected. 'Agriculture + wastewater' refers to groundwater sourced facilities likely to be influenced by agricultural activities and wastewater inputs to the environment. 'Wastewater' refers to groundwater sourced facilities likely to be influenced by wastewater inputs to the environment. 'Agriculture' refers to groundwater sourced facilities likely to be influenced by agricultural activities on the landscape.

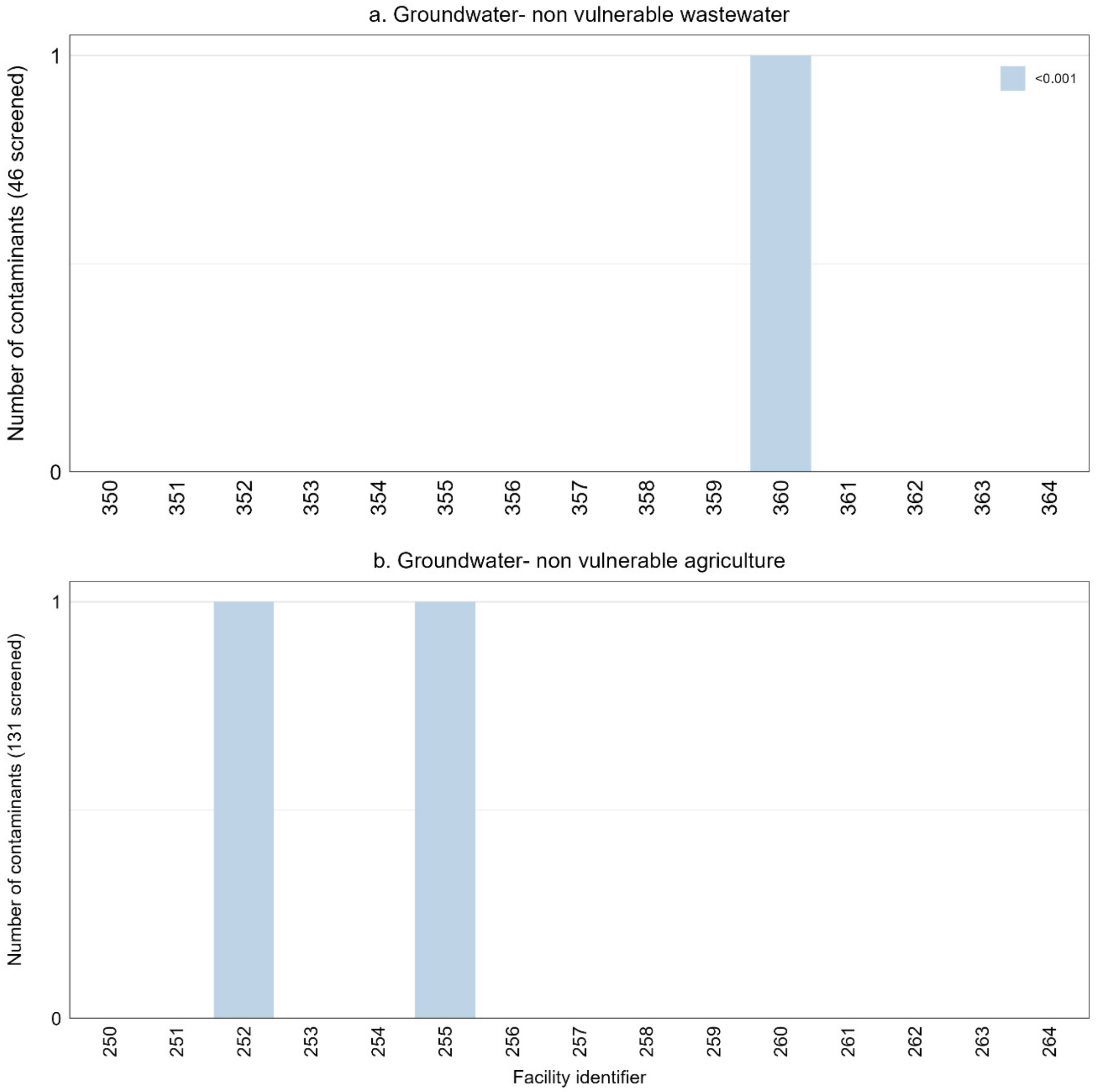

Figure S16. Bar charts showing the number of contaminants with exposure-activity ratios (EAR) that fall within specified ranges (see legend) in source and finished water samples collected from (a) non vulnerable groundwater sourced drinking facilities likely to be influenced by wastewater inputs to the environment, and (b) non vulnerable groundwater sourced drinking facilities likely to be influenced by agricultural activities on the landscape.
